# Membrane-Based Processes Used in Municipal Wastewater Treatment for Water Reuse: State-of-the-Art and Performance Analysis

**DOI:** 10.3390/membranes10060131

**Published:** 2020-06-25

**Authors:** Jiaqi Yang, Mathias Monnot, Lionel Ercolei, Philippe Moulin

**Affiliations:** 1 Laboratoire de Mécanique, Modélisation et Procédés Propres, Equipe Procédés Membranaire (EPM-M2P2-CNRS-UMR 7340), Aix-Marseille Univ., Europôle de l’Arbois, BP 80, Bat. Laennec, Hall C, CEDEX 04, 13545 Aix-en-Provence, France; jiaqi.yang@centrale-marseille.fr (J.Y.); mathias.monnot@univ-amu.fr (M.M.); 2 Société des Eaux de Marseille Métropole, 25 Rue Edouard Delanglade, CEDEX 06, B.P. 29-13006 Marseille, France; lionel.ercolei@eauxdemarseille.fr

**Keywords:** membrane processes, municipal wastewater reuse, disinfection efficiency, fouling, water recovery

## Abstract

Wastewater reuse as a sustainable, reliable and energy recovery concept is a promising approach to alleviate worldwide water scarcity. However, the water reuse market needs to be developed with long-term efforts because only less than 4% of the total wastewater worldwide has been treated for water reuse at present. In addition, the reclaimed water should fulfill the criteria of health safety, appearance, environmental acceptance and economic feasibility based on their local water reuse guidelines. Moreover, municipal wastewater as an alternative water resource for non-potable or potable reuse, has been widely treated by various membrane-based treatment processes for reuse applications. By collecting lab-scale and pilot-scale reuse cases as much as possible, this review aims to provide a comprehensive summary of the membrane-based treatment processes, mainly focused on the hydraulic filtration performance, contaminants removal capacity, reuse purpose, fouling resistance potential, resource recovery and energy consumption. The advances and limitations of different membrane-based processes alone or coupled with other possible processes such as disinfection processes and advanced oxidation processes, are also highlighted. Challenges still facing membrane-based technologies for water reuse applications, including institutional barriers, financial allocation and public perception, are stated as areas in need of further research and development.

## 1. Introduction

Traced back in history, wastewater has long been reused for humans directly or indirectly because of the water shortage and lack of treatment facilities. Since people realized that wastewater could cause serious catastrophic epidemics of waterborne diseases such as Asiatic cholera and typhoid, they started to research potable water protection and terminal wastewater treatment, such as the reservoirs or aqueduct systems and filtration [1]. The State of California in the Unites States (US) is a pioneer in promoting water reclamation and reuse for irrigation, and promulgated the first reuse regulations in the early 20th century [2]. At the end of the 20th century, the benefits of promoting wastewater reuse as a means of supplementing water resources have been recognized by most state legislatures in the US as well as by the European Union (EU) [1]. From then on, the evolution of processes for water reuse updates continuously with strict quality guidelines, which not only contributes to human health, but also protects the earth’s resources that humans depend. Nowadays, the term wastewater reuse is often used synonymously with the terms of wastewater recycling and wastewater reclamation. The US Environmental Protection Agency (EPA) defines wastewater reuse as: using wastewater or reclaimed water from one application for another application [3]. The deliberate use of reclaimed water or wastewater must comply with applicable rules for a beneficial purpose.

Water scarcity is growing under the pressures of population growth, climate changes and increased pollution. At present, one in nine people lacks access to improved sources of drinking water and one in three lacks improved sources of water sanitation [4]. From the Global Risks report of 2019 provided by the world economic forum, more than 650 million people in 500 cities are projected to face declines in freshwater availability of at least 10% in the 2050s, especially in Middle East and North Africa [4]. However, around 80% of overall wastewater is discharged into the world’s waterways. In this condition, water reuse is considered as a promising approach not only to purify the wastewater, but also to meet people’s needs. In fact, qualified reuse water not only reduces the occurrence and spread of diseases, but also contributes to the protection of the environment. Therefore, recovering water, energy, nutrients and other precious materials embedded in wastewater, is a key opportunity to be seized [5]. Water reuse could also help to regulate climate by increasing natural flows in surface waters (with cascading positive effects on ecosystem health and biodiversity) and may contribute to rising groundwater tables through water reused for crop or landscaping irrigation [6]. Moreover, increasing water availability through reuse may help to reduce conflicts over water due to scarcity or resource limitations.

Water reuse has become an attractive option for conserving and extending available water supply and its applications are shown in Table 1 [7]. Overall, water reuse plays a significant role in non-potable applications, occupying 97.7% of the water reuse market, including non-potable urban reuse, irrigation, recreation impoundment, environmental enhancements, industries and groundwater recharge. Mainly, the share of reclaimed water is mostly for irrigation, 52% in total, including agricultural irrigation and landscape irrigation [7,8]. Additionally, approximately 20% of reclaimed water is reused for the industrial self-water-circulation systems for resource and money savings. In order to treat wastewater for reuse as potable water, there are two main ways which are, respectively indirect drinking and direct drinking water. Indirect drinking water can be reused via surface water flow, surface water and groundwater recharge. Direct drinking water is the most contentious type of water reuse. Actually, the reused wastewater for drinking is always treated to a higher level based on more serious potable water criterion. However, the concerns of public health, water security, economic and equipment shortage are still barriers to people’s acceptance [7,8]. Thus, only 2.3% of reclaimed water is reused into potable ways.

As reported by Bixio et al. in 2005, over 3000 water reuse projects distributed in over 60 countries were assessed in an advanced planning phase. Most water recycling schemes are located in Japan (> 1800) and the US (> 800), then followed by Australia (> 450), Europe (> 200), the Mediterranean and the Middle East area (> 100) and Latin America (> 50). However, the projects number is likely to be higher considering the rapid development of water reuse in China, India and the Middle East [9]. Table 2. lists the current status of water reuse rates in different countries.

The USA stands out as it represents over one-fifth of the reused water capacity worldwide whereas it only provides a national water reuse rate of 9.7%. In the area with severe water shortage, for example the Arab Region, 18 out of 22 Arab countries fall below the water poverty line of 1000 m^3^ per capita in 2014. Driven by high pressure, 71% of the wastewater collected in the Arab States was safely treated, and 21% of them was being reused, mostly for irrigation and groundwater recharge in 2013 [10]. Moreover, Singapore has a population of over five million people with a total water demand of 1,700,000 m^3^·d^−1^. However, Singapore imported water from Malaysia to alleviate water shortages for a long period. The government extremely desires to be independent of water demand via water reuse.

Areas with high water consumption, such as China, are facing serious water shortages driven by rapid economic growth and urbanization according to its increasing population. According to the Chinese Ministry of Ecology and Environment, 73 billion m^3^·y^−1^ of wastewater was produced in 2015, but merely 2.9% of the total wastewater was treated to be reused. In Europe, the total reused water volume amounted to 964 million m^3^ annually, which accounted for approximately 2.4% of the treated urban wastewater effluents [13]. According to Hochstrat et al. [25], in terms of quantity, Spain accounted for the largest reused water quantity in Europe, about a third of the total water volume reused, followed by Italia, Cyprus and Malta. Furthermore, although wastewater reuse is in great potential, the reused water capacity seems rare compared with the total volume of municipal wastewater generated in the world, which was estimated to between 680 and 960 million m³ per day [6]. As reported by Global Water Market 2017, the total volume of water reuse was approximately 14.2 billion m^3^·y^−1^ worldwide, less than 4% of the total volume of wastewater [26]. Thus, the world has a high potential of establishing water reuse projects, improving water reuse qualities and reducing water crisis worldwide. Aimed at achieving water reuse, the essence is to purify sewage, removing pollutants and contaminants from wastewater. Therefore, it is necessary to correctly define the wastewater composition and target pollutants. Wastewater usually comes from households, schools, offices, hospitals, commercial and industrial facilities. Municipal wastewater or domestic sewage may have high levels of organic and inorganic material, pathogenic organisms, nutrients and many toxic elements, including heavy metals [27]. Generally, municipal wastewater contains approximately 99.9% water by weight [28]. The remaining 0.1% constituents in wastewater can be divided into several main categories presented in Table 3. Typically, untreated wastewater contains a variety of biologic and chemical constituents, such as bacteria, viruses and organic matters. Therefore, wastewater treatment is aimed at removing most of or all of, these constituents in sewage.

Finally, the reclaimed water should fulfill the criteria of health safety, appearance, environmental acceptance and economic feasibility, for reuse [29]. However, the components and pollutants in wastewater are changeable from source-to-source. Therefore, the treatment processes should vary with the components to be removed. Importantly, different reuse applications require different water quality specifications. Therefore, treatment technologies developed from simple processes into more advanced ones [30]. Since not all the wastewater treatment processes could remove contaminants from wastewater completely, the reused water needs to meet the water reuse standards formulated by the government or other qualified authority institutions that working for the applications or emission of effluents. However, there are no uniformly enforceable international water reuse guidelines to control the quality of the reclaimed wastewater. Even for the same water reuse application in the U.S., guidelines vary from states to state. Therefore, countries are looking forward to new regulations referring to international guidelines from the United Nations (UN), the EU Commission or the US EPA to estimate the different criteria needed for better water management [9,31,32].

Based on scientific consensus and best available evidence, the World Health Organization (WHO) has set some guidelines for the safe use of wastewater, excreta and greywater, promoting the public health benefits of wastewater, excreta and greywater use in agriculture and aquaculture and is now widely accepted as a benchmark [36]. Additionally, the European Parliament adopted a legislative resolution in 13 May 2020, on the Councils position at first reading, with a view to the adoption of a regulation of the European Parliament and of the Council on minimum requirements for water reuse (15301/2/2019—C9-0107/2020—2018/0169(COD)) [37]. This regulation project defines the water quality limits and obligations of wastewater treatment plant (WWTP) operators, for water reuse in the field of agricultural irrigation only. The above mentioned reuse guidelines, as the representatives concerning the parameters of water quality, are presented in Table 4. Actually, most countries supplement and develop their own guidelines, based on WHO guidelines—and considering their potential specifications—EU countries will adjust their reuse guidelines based on European water reuse requirements. Overall, the following parameters: pH, total suspended solids (TSS), biological oxygen demand after 5 days (BOD_5_), turbidity, total coliform and fecal coliforms are important parameters to evaluate the water quality all over the world, and they will be considered to evaluate the ability of some water reuse technologies in the following parts of this study.

To be reused, the reclaimed water should achieve reuse limits to ensure human health and environmental protection. The choices of wastewater treatment technologies may be affected by the following factors [38]: reclaimed water objectives for application, wastewater characteristics, compatibility with existing conditions, process flexibility, operating and maintenance requirements, energy and chemical requirements, economical requirements, residual disposal options and environmental constraints. In general, in a WWTP, if the wastewater, after primary and/or secondary treatment, cannot be reused for target purposes, it should be treated to tertiary level or with more advanced processes to achieve reuse standards.

Primary treatment can be considered as physical processes such as sedimentation and flotation [39]. The process can remove approximately 50–70 of total suspended solids (TSS) and 25–50 BOD_5_ [40]. The secondary treatment combines biologic and chemical processes to remove the soluble organic matter and the residual TSS after the primary process, such as activated sludge, oxidation ponds and rotating biologic contactor [41]. However, the secondary effluent is limited in reuse applications with risks for human health and public environments [42]. A tertiary treatment could then be applied to remove associated hazards and health risks [42]. Tertiary and advanced treatments are considered as the advanced level of treatment and mostly include membrane processes, advanced oxidation processes, disinfection or any combination of them to remove chemical and biologic constituents. The advanced processes remove most of the suspended solids, colloids, bacteria and viruses from the feed [43], nearly up to 99% removal of the contaminants, thus for safe water quality in specific reuse [44]. According to the classified treatments, the requirement for reuse should be stricter in the field of human exposure due to potential health risks. Where human exposure is likely, reclaimed water should be treated to a high level. In order to make reuse cost-effective, the level of treatment must be “fit for purpose”.

Treatment technologies are employed either alone or in combination to achieve wastewater reuse levels. However, as the potential for human contact increases, advanced treatment beyond secondary treatment becomes more accepted and membrane filtration is one of the key unit technologies for water reuse. Although several review articles have given overview summaries of the previously reported techniques for municipal wastewater reuse [45,46,47,48,49], they either mainly focused on greywater reuse or on only one reuse level or on only one type of technology. Therefore, membrane-based municipal wastewater treatment techniques for reuse have not been extensively reviewed.

This study aims to provide a comprehensive review on membrane-based processes used in municipal wastewater treatment for water reuse. Particularly, this review is expected to provide an overall summary of reported research cases of feasible membrane-based technologies on municipal wastewater treatment for reuse, mainly focusing on membrane hydraulic performance, contaminants removal ability, reuse purposes and energy consumption. Key benefits and challenges of current implementations will be also described after the summary.

## 2. Performances of Membrane-Based Treatment Processes for Municipal Water Reuse

Membrane-based technologies are considered to be integral units for municipal wastewater treatment processes [50]. Pressure-driven membrane processes are presented in Table 5 and can be classified into four main categories, based on the different selective pore size: two low and two high pressure processes microfiltration (MF) and ultrafiltration (UF), nanofiltration (NF) and reverse osmosis (RO), respectively [47,51,52,53,54]. As the pores get smaller, the membrane needs more driving force to be operated [55,56]. When it comes to membranes, membrane fouling always occurs during the filtration process, followed with an increase in transmembrane pressure (TMP) to maintain a constant flux or followed with a decrease in flux to maintain constant TMP. According to Guo et al. [57], membrane fouling is often defined based on the type of pollutants present in the feed stream and can be classified into four categories, including: (1) particulate/colloidal fouling, when suspended solids and/or colloids clog the pores of a membrane or adhere to its surface; (2) organic fouling, when reactive dissolved organic components or colloids attach to the membrane by adsorption; (3) inorganic fouling, also known as scaling or precipitation fouling, caused by the presence of crystallized salts, oxides and hydroxides in the feed solution that are prone to precipitate onto membrane surface due to pH change or oxidation; and (4) biofouling, caused when microorganisms, plants, algae or other biologic contaminants growing on or in the membrane surface and pores. Each of these types of fouling can be either reversible or irreversible and permanently compromise its performance. All the fouling types can take place simultaneously, and the interaction between them usually increases filtration resistance [58]. Therefore, in each membrane process, fouling issues need to be considered and optimized to obtain sustainable and excellent filtration performance. Additionally, an important notion to take into account is the threshold flux, the flux at or below which a low and near constant fouling rate occurs, but above that which the fouling rate increases markedly [59,60]. The number of studies on critical, sustainable and threshold flux of different kinds of wastewater filtrated by certain membranes are expanding recently because these concepts of flux are highly related to control low fouling rates on membranes [59,61,62]. Especially for commercial and industrial production, the operation at sustainable flux could definitely control fouling and at the same time, give an optimal balance between moderate operating costs and moderate capital costs [59]. Concerning membrane materials for wastewater reuse, there are commonly polymeric membranes or inorganic membranes [53]. The selection of material is affected by pore size distribution, wetting susceptibility, porosity, mechanical strength, cost, polymer flexibility, fouling resistance, stability, durability and chemical resistance [56].

### 2.1. MF/UF Treatment Process after Secondary Treatment in WWTP

MF and UF can remove particles larger than their pore size mainly through a sieving mechanism [56,67]. In general, the MF process alone can effectively remove high molecular weight organic matters, suspended solids, colloids, bacteria and thus reduce turbidity. Compared with MF, UF membranes have a wider separation range with smaller pore size and enhanced removal ability for particles, colloids, and more importantly, bacteria with high removal rate and viruses. In order to clearly understand the separation difference between MF and UF, Tchobanoglous et al. [55] quoted by the review of Warsinger et al. [47], summarized the rejection characteristics when filtrating the same secondary effluent by MF and UF, on bacteria, viruses and other essential water quality parameters such as TSS, chemical oxygen demand (COD) and TDS. It should be noted at first that the data gave general information with certain limitations: the actual performance may vary related to different conditions, such as temperature, flow rate and TMP. Through comparison, both UF and MF were in poor removal efficiency on TDS, NH_3_–N and NO_3_–N. UF is almost as efficient as MF in removing TSS and BOD_5_, with the removal rates from 95% to 99.9% and from 75% to 90%, respectively. Moreover, the removal efficiency by UF on COD and total organic carbon (TOC) concentrations was about 5% to 20% higher than MF. More importantly, UF provides almost complete removal of bacteria, protozoa and viruses, which is one of the main advantages compared to MF. As stated by Warsinger et al. [47], UF can support up to 6 log removal of bacteria and up to 7 log removal of viruses, and if there are protozoa, UF can remove protozoan cysts and oocysts to more than 6 log reduction. These removals are effective if the upstream concentration allows it. Although MF pore size seems larger than the size of the viruses, it can eliminate some of the viruses and protozoa through adhering to impurities in wastewater like protein, colloids or coliforms [68]. In 1995, Madaeni et al. [69] have verified that the presence of bacteria (*E. coli*) and turbidity highly contributed to the *poliovirus* rejection through 0.22 μm MF under TMP between 0.5 and 1 bar. Huang et al. [70] demonstrated that virus (MS2 bacteriophage as an indicator) is more likely to be removed through 0.1 μm-MF in waters with more organic matters or higher concentration of large molecular weight organic foulants. Herath et al. [71] successfully observed a considerably high virus rejection rate at their isoelectric point when using a 0.2 μm MF membrane to filtrate the specific virus (19 to 80 nm) solutions due to the virus–virus coagulation and virus–protein coagulation. In addition, the physicochemical properties of the virus, the membrane material, the feed, as well as the hydraulics of the filtration process could affect virus removal by MF [70,72,73]. For example, Huang et al. [70] recorded that when adding sodium or calcium to feed water, the virus removal capacity decreased immediately. Besides, low pH value increases virus removal while higher pH value makes removal rate decrease [71]. Anyway, the removal of viruses through MF is significant but not complete because the virus are still able to pass through membrane pores [74]. It is reported by Warsinger et al. [47] that the log removal value of MF processes on viruses ranges from 0 to 2 (equals to 0% to 99.9%). Table 6 summarizes the real cases of MF/UF processes applied alone after secondary treatment for water reuse. It can be seen that a few references demonstrate MF process alone to achieve water reuse. Although Ahn et al. [75] showed that MF permeate can be used to clean building floors or flush toilets, they did not state the water reuse standard that they referred to. When comparing their permeate quality with WHO guidelines, the detected parameters are incomplete and substandard, e.g., the turbidity in both cases exceeds the limit of WHO guidelines (turbidity ≤ 2 NTU). Normally, UF modules contain a prefilter as pretreatment (5–200 μm) to intercept large particles and improve UF performance by decreasing the formation of a cake layer on the membranes, resulting in a significantly reduced TMP and energy consumption [76]. While using UF membrane modules, it is more likely to achieve safety wastewater reuse on non-potable applications, such as agriculture irrigation and process water. According to Falsanisi et al. [77] and Muthukumaran et al. [78], it can produce qualified permeate that satisfies WHO water reuse guidelines when using UF process after conventional secondary treatment. However, both MF and UF have minor effects on the removal of residual nutrients such as phosphorus, nitrates and ammonium, but sometimes quite good effects on the removal of residual COD and TOC. Generally, effluents from WWTPs still carry high TSS and natural organic matters (NOM) which are prone to cause the formation of fouling on the MF/UF membrane easier. In fact, dissolved organic matters (DOM) cannot be removed effectively by MF or UF systems, but conversely it can be the main cause of fouling formation on the membrane, finally resulting in shortening membrane lifespan, reducing flow rate, increasing TMP and energy consumption [79,80]. Considering the hydraulic filtration performances (in Table 6), almost all researchers were interested in fouling control. Under constant permeate flux of 20 L·m^−2^·h^−1^, reported by Falsanisi et al. [77], the TMP on UF would increase fast from 0.3 bar to almost 1.2 bar in only 20 min filtration, and permeability decreased from 150 L·m^−2^·h^−1^·bar^−1^ to about 10 L·m^−2^·h^−1^·bar^−1^. While, according to Pollice et al. [81], with a periodic filtration cycle of the permeate (90–360 s) and a backwash of (30–40 s) in continuous operational process on secondary effluent treatment in autumn, the system could slow down the increase of TMP on UF which increased from less than 0.1 bar to about 0.7 bar in more than 30 days under almost stable flux at 20 L·m^−2^·h^−1^. Therefore, periodic backwashes could prolong the UF operation time, reduce the frequency of chemical cleaning uses and save energy during long term operation. Therefore, to support sustainably high performance of MF/UF on wastewater treatment in long term operations, it is necessary to conduct suitable physical and chemical cleaning on membranes.

In summary, direct MF and UF processes for the treatment of terminal municipal wastewater treatment provide recyclable water for non-potable reuse applications based on water reuse guidelines (Table 4). Because of the incomplete removal performance on bacteria, viruses or DOMs, the effluent after MF/UF system may have potential safety risks when exposed to humans and animals. In addition, when feed water contains much TSS, DOMs or other particles, the high fouling potential may cause severe damage to membranes, decrease production efficiency and increase the economic cost. Therefore, it is necessary to couple the membrane advantages with chemical, physical or biologic processes to enhance system performance, which is the topic of the next section. Moreover, MF/UF membranes are also widely applied in membrane bioreactors (MBR) or as the pretreatment for nanofiltration (NF) or reverse osmosis (RO) [82], as described in the following sections.

### 2.2. MF/UF Coupled with Chemical/Physical Processes after Secondary Treatment in WWTP

The hybrid chemical and physical processes with MF/UF methods such as sedimentation, adsorption, flocculation and coagulation [83,84,85]. Al_2_(SO_4_)_3_, Fe_2_(SO_4_)_3_, FeCl_3_ and polyaluminum chloride, have been developed widely and are used as remarkable coagulants [86]. Concerning adsorption, activated carbon (AC) is a widely accepted adsorbent [87]. It can be used as a powder (PAC) in dispersion or as granules (GAC) in fixed bed. Table 7 summaries the studies based on the combination of physical and/or chemical processes before or after MF/UF for wastewater reuse. Some literature used chemical or physical processes as post-treatment processes after MF/UF, most commonly used as a hybrid MF-adsorption system, such as MF–granular activated carbon system by Shanmuganathan et al. [88]. It was already verified that activated carbon, after membrane filtration, contributes to the additional removal of DOCs and trace organics, that are not completely retained by membranes [89,90]. The design of chemical and/or physical processes is mainly used as pretreatments before MF/UF to decrease membrane fouling potential and improve filtration performance [84,86,91]. Additionally, pretreatment processes support better filtration hydraulic performance for MF/UF. As stated by Zheng et al. [92], when studying the time required to increase TMP from 0.22 bar to 0.7 bar on secondary effluent treatment (constant flux of 50 L·m^−2^·h^−1^), direct UF process took only 12 h while the sand-UF system could extend to 30 days. In addition to that, Fan et al. [93] designed parallel tests of MF with/without pretreatment to treat secondary effluent under constant TMP (0.70 bar), the results confirmed that the coagulation-prefiltration (1.5 μm)-MF systems could improve flux recovery (J/J_0_) between 20% and 30% higher than MF alone due to the hydraulically irreversible fouling (internal adsorptive fouling) reduction by coagulation. Therefore, a pre-filtrated treatment process removes the fouled components before MF/UF membranes, thus contributing to prolonging the whole system operational time, improving permeability recovery, and decreasing membrane cleaning consumption. First, it should be noted that in some pretreated processes, for example coagulation with settling, the re-growth of floc could conversely cause severe cake fouling on membranes [94]. It is necessary to add another prefilter after coagulation, flocculation and settling to remove the flocs, colloids and other particles before the membranes. Second, it has been proven that pretreatment processes could effectively remove NOM and colloids [95,96]. As described previously, dissolved organic carbon (DOC) cannot be effectively retained by MF or UF due to membrane pore sizes being much larger than component molecules [97]. Fan et al. [93] reported that MF could only remove 3.9% DOC, while UF could remove 24.7%. Therefore, when pre-coagulated with 5 mg·L^−1^ of Al^3+^, the MF system could increase 10% to 15% DOC removal; and when pretreated with 10 mg·L^−1^ anion exchange resin in wastewater which removes primarily lower molecular weight cut-off (MWCO) and negatively charged organic fractions, the MF and UF systems could improve DOC removal to 58.8% and 68.3%, respectively [93]. Third, pretreatment processes contribute to the removal of dissolved nutrients, mainly nitrogen and phosphorus. Hybrid precipitation–MF system by Lu et al. [98] made use of calcium salt to increase precipitates involved with phosphorus and fluorine and then separated solid–liquid phases by following with crossflow MF for separation. Guo et al. [84] compared the performance of MF with and without pretreatment for wastewater reuse, the results showed that MF alone only removed 20% TOC and 5% PO_4_^3−^, while pretreated with flocculation and adsorption, the removal efficiency of TOC reached 99.7% and more than 97% of PO_4_^3−^. In addition, the UF system coupled with PAC can be used to eliminate both DOM and micropollutants. Though MF/UF alone is inadequate for micropollutant removal, in PAC–membrane processes, contaminants (including natural disinfection byproduct precursors) can be reduced through adsorption onto the activated carbon particles, which are then separated from water by either UF or MF [68]. Among MF-based treatments in Table 7, the hybrid granular active carbon with MF and coagulation with MF processes were performed with significant removal efficiency on turbidity, TSS and color [88,99,100]. More notably, these processes were able to remove between 40% and 46% of DOC from the feed, mainly caused by coagulation and adsorption, resulting in lower fouling formation and lower energy consumption [88,100]. However, based on the WHO water reuse guidelines, MF permeate is mostly applied in low levels of non-potable reuse, such as toilet flushing, machine cleaning, irrigation for non-food crops or more likely reused as process water for industries, such as washing, cooling and circulating. Among UF-based treatments in Table 7, the hybrid flocculation, coagulation or adsorption process with UF processes, could remove turbidity, TSS, color almost completely from secondary treated municipal WWTP. In addition, the removal of COD, DOC, metal and salt ions was also higher than MF. More important, UF could remove bacteria, viruses and parasites [101,102]. Therefore, hybrid UF permeate can contain little to no concentration of TSS, microorganisms, relatively low concentration of micropollutants, relatively high concentration of nutrients, and exhibits favorable inorganic ratios [103].

In fact, the permeate of UF as the main technology for reclamation is commonly reused on non-potable applications, such as agricultural irrigation, landscape irrigation, urban reuse, car washing and sometimes as process water for industries. However, the limitation for hybrid UF, with physical or chemical processes for potable reuse, may be the residual dissolved organic matters, micropollutants, and specific toxic ions produced from coagulants or flocculants (such as chloride ions). When pretreated with adsorption, the disadvantages include the expensive regeneration of the adsorbents (such as PACs) and loss of adsorbents [104]. Moreover, pretreatment with coagulation, flocculation and adsorption need higher energy cost with increased sludge volume production during treatment [105].

In summary, the use of MF or UF alone after conventional secondary treatment as well as hybrid MF or UF provide a relatively good quality of permeate compatible with non-potable reuse applications. Therefore, the membrane bioreactors (MBR), operated as secondary treatment in some recent WWTPs, could also be an interesting process for water reuse applications, which will be the topic of the next section.

### 2.3. MBR-Based Treatment for Water Reuse

Besides chemical and physical processes, biologic processes could also be combined with a membrane system based on its advantage in the degradation of microorganisms [110]. Alternatively, Membrane Bioreactor (MBR), a combination system, is mostly constituted with a conventional activated sludge process (CAS) and a submerged or external MF/UF membrane process. The CAS process is a biologic process that is mainly used for reduction of organic matters in the wastewater, and usually includes an aeration tank used for biologic degradation and a secondary clarifier (sedimentation tank), where the sludge is separated from the treated effluent [111]. The effect of the membrane is to increase the concentration in the biologic reactor, to retain the particulate phase within the bioreactor and allow permeate to pass to the next process or be discharged/reused [112]. In MBRs, biodegradation and membrane separation are performed simultaneously, thus maximizing wastewater treatment efficiency. Compared to conventional activated sludge with a membrane post-treatment process, MBRs perform better in processes producing high biodegradation efficiency, small footprint, smaller sludge production, good resistance to shock loading and automation capability [113]. This section does not aim to provide an exhaustive review of MBR used for water reuse as it has been the topic of numerous previous review papers, but it rather aims to give a comparison point for the other processes presented in this study. A review by Wu et al. [45] detailed the non-potable and potable application of various MBR systems for greywater reclamation with numerous MBRs case analysis. Normally, the wastewater generated from households or office buildings from streams without fecal contamination is called greywater, which is less polluted than municipal wastewater and easier to be treated for reuse. The reclaimed water is proved to satisfy the guidelines of US EPA and WHO reuse applications, such as flushing toilets, irrigation, washing cars, recharging aesthetically pleasing natural or underground water systems. It is reported that the MBRs performed better in the removal of organic matters, resulting in relatively less energy and economy consumption compared with conventional CAS processes. In addition, Wu et al. [45] and Atasoy et al. [114] both stated that greywater produced less organic foulant for MBRs together with the lower driving force and lower membrane fouling. It is important to note that (i) greywater used in these papers contains fewer organics, fewer bacteria and pathogens than municipal wastewater; (ii) the reported permeate fluxes of MBRs for greywater treatment were relatively lower compared to conventional centralized MBRs for municipal wastewater treatment (generally within a range of 15–30 L·m^−2^·h^−1^ for organic membrane) which resulted in a thinner density of fouling [113] and (iii) lower fouling potential on membrane results in lower energy consumption. Studies summarized in Table 8 are focused on MBRs for municipal wastewater treatment and reuse applications. The results indicate that the MBRs could effectively remove organics, TSS, nutrients in various forms, surfactants and micropollutants from various wastewater. MBRs have been reported to consistently achieve removal rates of 90–95% for COD, 80–99% for NH_4_–N and 70–99% for total phosphorus (TP), respectively [115]. Although feed characteristics among various municipal WWTPs showed a large difference, the results of organic removal ratios in the MBRs presented only slight dissimilarities. Apart from greywater reuse, black water (containing fecal matter) and domestic wastewater can also be reused under MBR treatment, which has been verified by several researchers such as Purnell et al., Atasoy et al. and Xing et al. [114,116,117]. The application of reclaimed municipal water can be in non-potable uses with MBRs or additional processes. In addition, MBRs are considered as the cost-effective technology for wastewater treatment which can be operated under a widely organic loading rate, high concentration of mixed liquor suspended solids (MLSS) and a large amount of feed flow. This view can be supported by Tam et al. [118] who described the separately MBR and MF processes on the same sewage treatment, when the water production both achieved the reuse level for toilet flushing, the MF feed was secondary treated effluent whereas the MBR feed was just degritted sewage. Nevertheless, MBRs are confirmed to produce high qualified water production even under higher concentration of COD, BOD_5_, TSS, TN, TP and turbidity in the feed. In general, municipal wastewater after the MBR treatment process has been usually reused as toilet flushing [119,120,121]—and some even can be reused for irrigation [122]. Studies have demonstrated that MBR treatment removed different types of microorganisms, including enteric viruses in some studies, more effectively than in conventional secondary treatment, performed on both pilot-scale and within full-scale municipal WWTPs [112,123,124]. It is important to note that the removal value is a function of the inlet concentration and outlet after MBR. As stated by De Luca et al. [125,126], the MBR process was able to achieve, respectively 2.7 and 1.7 higher log reduction value (LRV) of somatic coliphages and F-RNA specific bacteriophages compared to CAS process. Similarly, it was reported by Francy et al. [123] that LRV for all organisms, including enteric viruses, were higher by MBR treatment (3.0 to > 6.7) than by conventional secondary treatment (1.5 to 4.2) in municipal wastewater treatment. Hirani et al. [127] compared six MBR systems with membrane pore sizes ranges from 0.03 to 0.1 μm to evaluate their removal capability on bacteria and phages. The results showed the LRVs of coliform bacteria, fecal coliform bacteria and indigenous MS-2 coliphages with ranges of 5.8–6.9, 5.5–6.0 and 2.6–3.4, respectively, were in minor difference among different MBR systems. In addition, Chaudhry et al. [128] demonstrated the MBR process could provide more than 4-log reduction of adenovirus and norovirus GII, and over 5-log reduction of F^+^ coliphage, and provided evidence for assigning virus disinfection credit to similar MBRs for reclaim wastewater [128]. The above cases confirmed the stable effectiveness of MBRs on certain bacteria removal whatever the pore sizes, but it is not for all microorganisms. Additionally, Hirani et al. [127] also reported the removal efficiency of seeded coliphages among different MBRs, the LRVs ranged greatly from 1.0 to 4.4 which is correlated to the membrane pore size distribution from 0.1 to 0.03 μm to some extent. For the same purpose, other studies draw a similar conclusion on the removal of seeded viruses in MBRs with LRVs ranging greatly from 0.4 to 5.8 with membrane pore sizes ranging from 0.1 μm to 0.4 μm [129,130]. However, size exclusion by the membrane is probably the dominant removal mechanism for bacteria and viruses in MBRs with intact membranes, but not exclusively. Other removal mechanisms, such as adsorption to the biomass, pore blocking and pore constriction may also play an important role [127]. Although the size of viruses are smaller than microfiltration pores, it is possible to achieve high removal rates of them mainly after the buildup of a biofilm on the membrane, which is in the same removal mechanism of MF mentioned above [124,131].

Furthermore, the effective biodegradation integrated with membrane performance leads to more advantages than separated processes, i.e., less organic foulants on the membrane, smaller footprint, faster plant activation, no biologic sedimentation units and less sludge production [125,132]. As a result, (1) particle separation can be achieved without sedimentation before MBR systems, but with membrane filtration [126]; (2) sludge production from MBRs is less than CAS [58,115]; (3) MBRs can provide footprint savings due to a higher organic loading rate and greater reactor depth compared to CAS. Membrane fouling remains a major obstacle that hinders faster commercialization of MBRs due to its influences on flux decline, membrane lifetime span, backwash frequency, energy cost, and even permeate quality. Generally, membrane fouling in MBRs should be blamed for both membrane pore-clogging and sludge cake deposition on membranes which is usually the predominant fouling component [133]. In fact, the complex nature of membrane foulants and activated sludge is the culprit to various fouling formation, such as cake fouling, pore blocking and extracellular polymeric substances (EPS) or soluble microbial products adsorption on/within membrane surface [134]. To control fouling, aeration is effective to remove cake fouling thus enhances the membrane flux, especially for submerged membrane [113,135,136,137,138]. But high aeration also brings impacts on biomass characteristics, since colloids and solutes would be the major membrane foulants which cannot be removed effectively by increasing shear stress [139]. In addition, since irreversible fouling still increased by physical washes (permeate backwash, air bubbles) directly influences the long-term operation of MBRs, chemical cleaning is necessary during the filtration process [134]. However, chemical cleaning should be limited to a minimum frequency when it can still maintain a sustainable operation of MBRs, because frequent chemical cleaning may lead to membrane damage, chemical agents’ consumption and environmental pollutions. In summary, MBRs provide qualified permeate for water reuse, while improvements need to be found to decrease membrane fouling and the related energy consumptions. In this case, it is necessary to find new anti-fouling methods for the MBRs. For instance, a novel magnetically induced membrane vibration system has resulted in higher flux at lower fouling rates compared with conventional submerged MBRs [140,141]. The vibration MBR was found to be a promising strategy in efficient fouling control and significant energy savings in future research.

More recently, anaerobic MBR (AnMBRs) are getting increasing attention and interest. Unlike MBR, AnMBR consists of a primary anaerobic bioreactor and a secondary MF/NF membrane bioreactor. First, the microorganisms in the anaerobic bioreactor convert organic carbon and BOD_5_ in wastewater into biogas (methane) and CO_2_. Second, membranes separate the microorganisms and other suspended solids from the treated effluent. The main advantage of wastewater treatment by AnMBRs is the natural aspects of the bioprocess such as the reduction of the overall energy demand, wide loading rate decreasing the need for aeration, increasing energy recovery from methane production and producing less secondary sludge [48,142,143]. These features create eco-environmental effects and supplement the energy cost during wastewater treatment. However, as AnMBRs become increasing popular on wastewater treatment, few cases have been investigated on whether AnMBRs alone can be used for wastewater reclamation. Once Song et al. [48] tested the performance of AnMBR on synthetic domestic wastewater in lab, the results showed that although the effluent after AnMBR achieved the high removal efficiency on COD (98.4 ± 0.4%), TOC (98.7 ± 0.3%) and TN (20.4 ± 11%), the quality cannot meet reuse standards due to the high concentration of COD (101.5 ± 22.9 mg·L^−1^) and TN (132.5 ± 16.9 mg·L^−1^) in the effluent. It showed the water production may contain more organic and inorganic matters from the AnMBRs than from MBRs. In addition, it has been reported that AnMBRs caused serious membrane fouling potential with high mixed liquor suspended solid (MLSS) concentration [143] and need longer biomass retention time to support the slow-growing anaerobic microorganisms than aerobic MBRs [144]. To improve the hydraulic performance of AnMBRs, some articles found several solutions. For example, the anoxic-aerobic MBR system which separates the anoxic process and MBR process is helpful in the improvement of permeate quality together with energy saving [118]. According to Liu et al. [145], low TSS in the feed water, such as mixed liquor, contributes to a significant reduction of gas sparging demand for membrane scouring in an AnMBR. More interestingly, when combining AnMBR with the further zeolite–RO process by Li et al. [146], the permeate can finally reach potable reuse level. Therefore, further treatment process after AnMBR, such as physically adsorption, precipitation, advanced membrane filtration or disinfection, is necessary to control the fouling potential and increase the treatment efficiency of AnMBRs, when applied to water reuse processes [145] and the performance of some AnMBRs with a further treatment step, will be described in the next section.

These three sections showed that after UF/MF combined with secondary treatment or after MBR processes, water reuse is feasible for non-potable applications mostly. Consequently, for more advanced reuse applications, complementary treatments such as NF, RO or forward osmosis (FO) are often needed, and these will be the subject of the following Section 2.4 and Section 2.5. In addition, the combination of MF/UF with disinfection or advanced oxidation processes are also recommended for an improvement on water production safety, described in Section 2.6. In addition to the mentioned processes, additional posttreatment process after MF/UF, such as electrodialysis [147], can also be applied on wastewater treatment for reuse. However, it is rare to apply these combinations on municipal wastewater reuse because of the complex operational procedures, cleaning processes and difficulty to achieve cost-effective results based on the current references.

### 2.4. NF/RO/FO-Based Treatment Processes for Water Reuse

Nanofiltration (NF) or reverse osmosis (RO) have been recognized as an effective means providing safe and reliable source of water supplies for both potable water and non-potable water purposes [79]. NF/RO technologies are outstanding in wastewater reuse applications, especially in potable reuse levels, because NF/RO can eliminate effectively organic micropollutants which are the most concerns by humans, such as endocrine-disrupting compounds, pharmaceutically active compounds, pesticides, disinfectant by -products, trace organics, etc. [151,152]. In addition, the processes of NF or RO are both highly capable of reducing turbidity, TSS, color, COD and TOC completely from the feed water. In addition, NF/RO processes also show significant removal efficiency on conductivity, TDS, alkalinity, salinity, hardness and ions from the feed which can be of particular interest for irrigation and potable purposes. Due to the difference of membrane resistances, the pressure driving force and energy costs are lower for NF compared to RO. In addition, forward osmosis (FO) is a promising membrane technology in the wastewater treatment field using a similar membrane to RO or NF membranes, but an osmotic pressure difference as a driving force [153,154,155]. Like the RO membrane, the FO membrane can guarantee a high rejection rate of solute, heavy metal and micropollutants [156,157]. Detailed information on NF, RO and FO for municipal wastewater reuse will be provided in the next paragraphs.

For NF removal efficiency, the separation characteristics of NF include pore-size steric mechanism (convective flow), solution–diffusion mechanisms and electrostatic interactions [63]. In addition to the substances that can be removed by MF/UF, high removals of constituents such as dissolved solids, dissolved salt ions, organic carbon, inorganic ions, and other organic compounds can be achieved by NF due to tiny pore sizes [45,158]. NF especially provides high rejection of divalent and multivalent ions, such as sodium sulfate, while it also permeates parts of monovalent ions (such as sodium chloride) through the membrane [63]. For example, Dalar et al. [159] demonstrated that the coupled MBR and NF system could remove almost all divalent and multivalent ions, such as PO_4_^3−^ and SO_4_^2−^, but only removed less than 60% monovalent ions, such as NO_3_^−^, Na^+^ and Cl^−^. Similarly, Bunani et al. [160] reported that the permeate produced by different NF membranes both contained less than 10 mg·L^−1^ divalent ions (Ca^2+^, Mg^2+^ and SO_4_^2−^), but contained concentrations higher than 206 mg·L^−1^ for Cl^−^, 134 mg·L^−1^ for Na^+^, 31 mg·L^−1^ for HCO_3_^−^. However, if comparing the Ca^2+^ removal efficiency by NF in Table 9, as in the work of Dolar et al. [159], the removal efficiency of Ca^2+^ seems less compared with other divalent ions, such as SO_4_^2−^ and Mg^2+^. Actually, ion rejection by NF membranes depends on the combination of electrostatic and steric interactions associated with charge shielding, Donnan exclusion and the degree of ion hydration [161]. In addition, rejection mechanisms by NF highly depend on membrane properties, physicochemical properties of solutes and feed characteristics (such as ions content, pH, organic matter concentration, etc.).

Whereas RO was originally conceived of as a method of producing potable water from the sea or brackish sources from the 1960s, the technology now is also widely applied on a large scale in municipal wastewater treatment and reuse [162,163]. The rejection mechanism of RO includes size exclusion, charge exclusion and physical–chemical interactions between solute and solvent [164,165]. As expected, RO retention capacities are better than NF due to its almost nonporous nature, reflected in higher removal of organic compounds, monovalent ions, TDS and conductivity [159,166,167]. As listed in Table 9, the conductivity removal by NF is usually effective, but not completely, ranging between 40% and 90%, while RO shows excellent separation performance on almost complete salinity removal. For example, Falizi et al. [168] designed parallel filtration tests with both NF and RO in order to find a more optimized method to reuse wastewater on agricultural irrigation, the results proved that RO could remove conductivity, TDS and various ions significantly while NF showed relatively lower removal efficiency. In addition, Shanmuganathan et al. [88] selected several NF and RO to filtrate the microfiltered and biologically treated sewage water. NF alone on monovalent ions removal could only reduce < 5% of NO_3_^−^, < 20% of Cl^−^ and Na^+^ from the feed, while RO showed high rejection ability on monovalent ions with > 88% removal of all ions. Similar removal results were also obtained for micropollutants, such as pharmaceuticals and personal care products (PPCPs) of which the RO process showed a wide range of higher rejection rate (from 10% to 60%) than NF [88]. The same results could also be proved by Gündoğdu et al. [169]. However, it should be noted that some NF and RO membranes materials are selective in removing micropollutants probably related to their rejection mechanisms [170]. For instance, the removal efficiency diminished considerably for micropollutants with a neutral or positive charge, of which the removal through RO was near 100% for naproxen, versus 20% for acetaminophen (neutral) and 60% for atenolol (positive) [171]. In addition, Simon et al. [172] assessed the adsorption of ibuprofen by NF and RO membranes was directly linked to the electrostatic repulsion among the pollutant, membrane and the solution’s pH. According to Shanmuganathan et al. [88], the diffusion phenomenon degrades the membrane’s efficiency in removing substances by adsorption.

However, due to their tiny and/or nonporous characteristics, the requirements of feed water for NF and RO membranes are relatively higher than MF and UF, because even small particles such as the NOM and submicron particulates in the feed are prone to cause severe fouling problems on these membranes [173,174]. In particular, biofouling, the irreversible adhesion on a membrane caused by microorganisms and the extracellular polymers, has been considered as the main obstacle during long term NF/RO operation [174,175,176,177]. In order to improve filtration performance, pretreatment processes ahead of NF/RO are required to reduce the potential of fouling development [79,178]. Pretreatment can prolong membrane lifespan and save costs, with the capacity to remove more organic compounds comprised of the soluble microbial products and extracellular polymeric substances. Conventional pretreatment processes before NF or RO consist of sedimentation, clarification, coagulation and flocculation, scale inhibition, activated carbon adsorption, dual media filter or dissolved air flotation [179]. However, it is more common to apply non-conventional pretreatment processes ahead of NF/RO, such as MF, UF and MBRs [177,180]. Some review papers [79,180] suggested that the non-conventional pretreatments were more effective than the conventional pretreatments for producing better water quality, despite biofouling attack and for minimizing the overall treatment cost. Notably, before NF/RO processes, the pretreatment with MF, UF, MBRs would be more efficient for fouling control than with cartridge (~10 μm) pretreatment which enable colloids and suspended particles to pass through membranes [181]. Table 9 summarizes the NF/RO-based membrane treatment studies applied to municipal wastewater reuse and most of them using non-conventional pretreatment processes before NF/RO. For example, MF could definitely provide better water quality improvement together with expenditure saving compared with lime pretreatment, as MF can reduce microbial contamination thereby reducing the rates of fouling and biofilm formation in subsequent RO [68]. Municipal wastewater treatment by hybrid pretreatment with NF/RO processes improves the produced water quality to potable reuse level. For instance, the hybrid MBR with NF or RO treatment system could both significantly remove all organic compounds investigated in this study with over 95% removal efficiency for all, thus producing an adequate permeate for indirect drinking water reuse [182]. Similar results were also reported by Jacob et al. [183] with the combination of MBR and NF/RO system and by Garcia et al. [152] with the combined MF and NF/RO system. Despite the fact that MBR is most commonly designed with MF or UF membranes, an innovative MBR designed with NF membrane was used before RO in the work of Tay et al. [184], the results showed that NF–MBR process provided higher organic/inorganic removal and the calculated energy consumption proves the feasibility of the NF–MBR+RO system for higher recovery water reclamation, compared to UF–MBR+RO system. This will be further discussed in Section 2.5. Besides having the advantages of energy recovery by AnMBRs, as stated in Section 2.3, the combination of AnMBR and RO systems could achieve water reuse with direct energy recovery from municipal wastewater with minimized sludge production and significant energy savings [145,185]. As technology advances, innovative technologies occur in the pretreatment field to enhance filtration performance and overcome difficulties for specific conditions, as for example in Singapore. Gu et al. [185] proposed an AnMBR+RO+ion exchange (IE) system to treat raw municipal wastewater for NEWater production. Here, the NEWater represents the high-grade reclaimed water produced from treated used water that is purified further using advanced membrane technologies, making the water ultra-clean and safe to indirect potable reuse in Singapore [22]. In this process, AnMBR removed 95.6% influent COD and converted 76.8% of COD to methane equivalent to 48% of the total energy the system consumed. RO rejected more than 95% of organic carbon, ammonium, phosphate and major ions from AnMBR effluent. Finally, IE was performed as a further polishing step mainly to remove residual ammonium under 1 mg·L^−1^ to meet the NEWater quality requirements for industrial and indirect potable use. This proposed process was estimated to reduce 68.3% of net energy consumption compared to the current process (secondary treatment + MF + RO) for NEWater production, together with a significant space saving [186].

Forward osmosis (FO) also called as direct osmosis is a new membrane-separation process in which water moves spontaneously across a semi-permeable membrane from the feed solution (lower osmotic pressure) side to the draw solution (higher osmotic pressure) side [157,187]. Different from the pressure-driven membranes, FO is an osmotically driven technology operated at very low or even non-hydraulic pressure during wastewater treatment [188]. Compared to NF/RO, FO membranes have a lower membrane fouling potential due to loose formation and less compaction of cake foulants in the absence of hydraulic pressure [189]. When fouling occurs, it is readily reversible compared to pressure-driven membrane processes [190]. Kwan et al. [191] compared the biofouling formation on FO and RO with the same feed water, the results showed that FO biofilms grew in a loosely organized thick layer with lower hydraulic resistance to water flow and slower flux decline, while RO biofilms grew in tightly organized layer with a larger amounts of EPS per cell resulting in greater biofilm-enhanced osmotic pressure and hydraulic resistance to water flow. FO membranes present high contaminant rejection rates, high flux recovery after cleaning and high water recovery using low-grade energy resources [192,193]. However, one of the key factors on FO performance is the choice of an appropriate draw solution which should be characterized by high osmotic pressure generation, inexpensive, non-toxic to the environment and efficient regeneration [194]. It should be noted that when applying FO processes to wastewater treatment, further treatment is required to simultaneously separate product water for reuse and recover draw solution [195,196]. One of the most common combinations is the hybrid FO-RO system, in which the inlet wastewater firstly diffuses into high concentrated draw solution through the FO membrane, and then the diluted draw solution will be filtered by RO to separate water production and continuously reconstitute the draw solution [192]. In the hybrid process, FO is likely performed as a high-level pretreatment process which produces minimum fouling on RO than the other non-conventional pretreatments. The other combinations include hybrid forward osmosis with membrane bioreactors (FO–MBR), membrane distillation (FO-MD) and nanofiltration (FO–NF) which are promising technologies for wastewater reuse [197]. FO–MBR, also called osmosis membrane bioreactor (OMBR), is a newly emerging technology for water reuse in recent years. OMBR combines activated sludge treatment with FO membrane separation, usually followed by a post-treatment, such as RO [198]. In OMBR operation, feed water is extracted from the mixed liquor into the highly concentrated draw solution by the forward osmosis (FO) process, then organic matters and bacteria can be retained in the bioreactor and thus increase their biodegradation during operation [190,199]. FO–AnMBR system can produce methane during wastewater treatment, with conversion rates probably between 0.2–0.3 L_CH4_. g_COD_^−1^ in lab studies. [195]. This methane production can act as an energy supplement during wastewater treatment processes. Membrane distillation (MD) is an effective process for draw solution reconcentrate and permeated water recovery after the FO process, especially for high salinity separation from feed wastewater [189,200]. As reported by Husnain et al. [201], the FO–MD system could be operated at a stable and equal water flux for both FO and MD membranes over long-term experiments, together with more than 3 logs (> 99.9%) removal of ammonium, COD, arsenic and combined solutes in both synthetic and real wastewaters. According to Corzo et al. [197], a hybrid FO using magnesium chloride as draw solution with NF system successfully produced reused water for agricultural irrigation with stable permeability and low membrane fouling during long term operation. However, the weakness of the system was over 40% higher energy consumption than a traditional UF-RO system, mainly due to draw solution loss. Giagnorio et al. [202] proved the potential implementation of a hybrid FO–NF system on wastewater reuse, with a 85% water recovery. Interestingly, when using seawater as draw solution, it is possible to make desalination and wastewater treatment at the same time [196,203]. For example, a FO–RO system stated by Volpin et al. [193] used seawater as a draw solution to filter secondary effluent which led to reduced seawater osmotic pressure, thereby reducing the operating pressure of the RO to desalinate the diluted seawater. This FO–RO hybrid process achieved both wastewater reclamation and seawater dilution. Another popular process, OMBR–RO, is also recommended for this purpose. Compared to conventional MBR–RO, Luo et al. [190] confirmed that OMBR–RO process not only prevented the downstream RO process from severe membrane fouling, but also reduced the need for RO concentrate disposal, thus reducing energy consumption for seawater desalination and wastewater recovery. From the economical assessment, FO supports lower costs during treatment. For example, Cornelissen et al. [198] calculated a 18–23% cost savings by OMBR–RO system compared by conventional MBR–RO for wastewater reuse, with the following operating conditions: a flux of 20 L m^−2^ h^−1^ at TMP of 0.1 bar on MBR and a flux of 15 L m^−2^ h^−1^ at TMP of 14.5 bars on RO (MBR–RO); a flux of 20 L m^−2^ h^−1^ at 0.5-M NaCl on FO and a flux of 30 L m^−2^ h^−1^ at TMP of 31.7 bars on RO (FO–RO). Therefore, lower desalination energy demand and lower water augmentation can be achieved while delivering safe water for direct potable reuse due to the double dense membrane barrier protection.

Compared with the MF/UF/MBR based treatment processes, there are more research articles on wastewater reuse focusing on NF/RO processes. After all, the choice of a membrane system should be based on feed water quality and its application purpose to forego unnecessary expenses. For example, because NF or RO membranes have high removal ability on alkali, salinity and other organic and inorganic ions, the direct permeate decreases the soil permeability, loses pH balance and nutrients balance necessary for plant growth which is not a positive for irrigation reuse [204]. In this situation, many studies blend NF/RO permeate with less treated wastewater to produce water viable for irrigation and improve the reuse rate of treated wastewater at the same time [168,204,205]. Although FO is developing fast recently, there are not many related reports and cases on the application of municipal wastewater treatment and reuse. One of the key challenges to accomplish sustainable water recovery in FO is the separation and recovery of draw solutes, which accounts for most of the energy consumption [188]. In future research, the regeneration of draw solutes should be accomplished without using energy-intensive processes like RO. In addition, researches should also focus on the ideal membrane material, the mechanisms of membrane fouling and the cleaning strategies.

### 2.5. Membrane-Based Treatments with Disinfection and Advanced Oxidation Processes for Water Reuse

Although membrane separation processes are available to produce qualified permeate at a relatively high level, even to potable reuse, the health risks still exist, because micropollutants are not always completely removed and microorganisms can regrow during long-term transportation of water. In this condition, disinfection and advanced oxidation processes both as advanced technologies, are significantly effectively used as a supplementary process with membrane separation to eliminate the non-rejected micropollutants and microorganisms completely and in a remanent manner.

Disinfection is the essential treatment for healthy water reuse by reducing TOC and inactivating or destroying the pathogen microorganisms. The disinfection of water is usually carried out by physical processes (such as UV) [209] or by chemical disinfectants or sanitizers (such as chlorine, chlorine dioxide, hydrogen peroxide, ozone and chloramines, etc.) or by a combined process. Although membrane separation processes can reduce the microbial pathogens greatly, it is necessary to set up a disinfection process for terminal and remanent treatment to ensure safe levels of pathogens in case of external pollution of the effluent. The commonly used disinfection processes and their general performances are listed in Table 10. Among these approaches, chlorination is considered as the most common disinfectant in the form of gaseous chlorine, chloramines and sodium hypochlorite [210]. Chloramines are moderately effective on the inactivation of bacterial pathogens, while gaseous chlorine and sodium hypochlorite are both effective on the inactivation of bacterial and viral pathogens through generating free chlorine. Ozonation and UV processes are identified as more robust disinfection alternatives when used against protozoa, although ozone even has limited efficacy against Cryptosporidium [211,212]. According to De Sanctis et al. [213], although the investigated disinfection processes (UV and peracetic acid) had effective results for *E. coli*, somatic coliphages and total coliforms, they were both ineffective on *C. perfringens* spores conversely. Furthermore, the disinfectants used in chemical disinfection are relatively strong oxidants that can transform organics to disinfectant byproducts (DBP) [214]. The DBPs of the chlorination process are quite complex and variable. Chloramine is similar to chlorine gas and chlorine except for cyanogen chloride. In contrast, UV radiation is a non-byproduct process, and the DBPs of chlorine dioxide are less complex than chlorination processes, which usually produce chlorite, chlorate and chlorides. The selection of disinfection methods should be associated with influent characteristics, effluent quality, disinfectant agent toxicity, byproduct formation, cost and the final applications [211].

Disinfection helps to improve removal efficiency on microorganisms that cannot be eliminated by membrane processes, mainly MBR and MF or provide a disinfection remanent effect. Many cases of these combinations have been performed on water reuse applications, as showed in Table 11. In the combination of MBR with a post chlorination process described by Franca et al. [125] in municipal wastewater treatment, the chlorination of the permeate, after MBR, permitted further improvement in microbiologic characteristics, but without any variation on its physicochemical parameters. In practical applications, the managers always want to use disinfectants, mostly chlorination agents, with optimization on maximizing microorganism inactivation while minimizing the formation of DBP that are challenging to remove in the advanced treatment train [215]. A new sequential chlorination process using short-term free chlorine and chloramine has been developed on wastewater reuse applications, resulting in quick microorganism inactivation by free chlorine, low DBPs production and long-term residual retention by chloramine [215,216,217]. Similarly, the disinfection process sequential UV with chlorine is also proposed on the wastewater reuse application. As reported by Wang et al. [218], the treated permeate, after sequential UV/chlorine combination, could reduce the dose of chlorine, and decrease the genotoxicity from MBR–MF permeate, when compared to chlorination alone. With pretreatment of 4 mJ·cm^−2^ UV, 1 mg·L^−1^ chlorine was sufficient to inactivate bacteria under the undetected level, while 5 mg·L^−1^ chlorine was needed for complete disinfection without UV dose. Therefore, increased UV dose leads to stronger damage to bacteria, which is beneficial for chlorine disinfection efficiency with lower chlorine concentration. In addition, the combination of various disinfection processes has attracted increased attention, because the combination would include a wider range of pathogens, improve reliability through redundancy, reduce DBPs and save potential cost. For instance, it was found by Haaken et al. [219] that electrolysis can prevent reactivation of bacteria, after UV irradiation, through electrochemical production of oxidants in biologically treated wastewater. As a result, the hybrid UV disinfection, with the electrolysis unit, could ensure a reliable, sustained bacterial reduction and prevent the reactivation of reversibly damaged coliforms. In addition, Liberti et al. [220] recommended the combination of H_2_O_2_/Ag^+^ to be applied as an advanced, long acting residual disinfectant for high quality effluents compared with H_2_O_2_/Cu^2+^. The bactericidal activity (*E. Coli* tested) by a combined H_2_O_2_/Ag^+^ system could be additive—and improved with a pH and temperature increase—while the combination of H_2_O_2_/Cu^2+^ decreased both bactericidal and viricidal effects significantly because of the complexation of copper by organic matter when applied to tertiary effluent.

Advanced oxidation processes (AOPs) have been widely demonstrated to be reliable for wastewater treatment since they have a high capacity to oxidize nearly all organic pollutants mainly through the generation of hydroxyl radicals (OH•) or SO_4_^2^^−^ [221]. Based on the review by Silva et al. [170], AOPs have been demonstrated to be highly effective in micropollutant removal. In fact, if the disinfection processes described in Table 10 are considered as primary disinfection, AOPs can be considered as secondary disinfection or post-treatment disinfection [222]. Because some AOP designs can also play a role as disinfection, these AOPs become an integrated solution to some water quality problems [223]. For example, ozonation treatment of wastewater is well-known and used for both disinfection (taste and odor regulation, discoloration, germs and microorganism destruction) and oxidation (removal of micropollutants). Therefore, the application of AOPs stand out in degradation of micropollutants in both raw water and wastewater as well in the degradation of byproducts from primary disinfectants and in high oxidation potential [224,225]. The oxidation mechanism of AOPs include 2 steps of oxidation: toxic pollution in feed is firstly oxidized into biodegradable compounds, then oxidized into nontoxic compounds, such as water, carbon dioxide and inorganic salts. The realization of AOPs commonly depends on separate or various combinations of commonly used ozone (O_3_), hydrogen peroxide (H_2_O_2_), catalysts and ultraviolet light (UV) to produce •OH under mild conditions (low temperature and pressure) [47]. Some other AOPs depend on ultrasonic irradiation, electrohydraulic cavitation, X-ray or gamma-ray radiolysis [222]. According to AOP-related review articles, photolysis processes, ozone-based AOPs including ozonation, O_3_/UV, O_3_/H_2_O_2_ and O_3_/H_2_O_2_/UV, hydrogen peroxide-based methods (i.e., H_2_O_2_/UV, Fenton, Fenton-like, hetero-Fenton and photo-Fenton), heterogeneous photocatalysis (TiO_2_/UV and TiO_2_/H_2_O_2_/UV systems) and sonochemical and electrochemical AOPs (EAOPs) are the commonly used as single or combined types for AOPs in wastewater treatment [226,227]. Beyond the summaries, the innovative solar driven AOPs are attracting more attention recently, such as H_2_O_2_/sunlight, TiO_2_/sunlight, H_2_O_2_/TiO_2_/sunlight, natural photo-Fenton [228]. All of these showed possibilities in the purification of effluent from urban wastewater plants for reuse. From these AOPs, ozonation, UV/ozone, UV/hydrogen peroxide and photocatalysis are most frequently studied and utilized for many applications. Based on this information, the aim of this review is to report the applications, and the results that advances of AOPs in wastewater treatment technologies have produced, with the purpose of providing insight towards the application of such technologies for efficient water reuse.

Currently, the combination of membrane filtration with AOPs is advantageous in wastewater treatment since it enables us to permanently solve or alleviate the problems of membrane fouling, to remove more microorganisms and micropollutants from water—and to reduce the disposal of generated concentrates during membrane separation [229]. Table 12 shows the performance of membranes combined with AOPs on wastewater reuse applications. The combination of AOPs membrane filtration for municipal wastewater reuse are typically in two types: (a) pretreatment process to degrade organic compounds in the membrane feed stream; (b) posttreatment process for mineralization of non-rejected micropollutants in the permeate stream [226]. The use of AOPs on membrane concentrates to reduce the discharge problem could also be an interesting option but has not been studied yet in the field of municipal wastewater reuse. AOPs as the pretreatment step before membrane processes are usually aimed at controlling membrane fouling potentials through oxidation of organic matters and micropollutants. As described by Qi et al. [230], an integrated ozone-biologic aerated filter system was shown to provide qualified effluent before the posttreatment by UF–RO during wastewater treatment, which contributed to improving the end permeate quality, decreasing membrane fouling and decreased energy consumption. Indeed, when combining ozone with the biologic method in wastewater treatment, ozone contributes to removing more COD, color, pathogens and increasing the biodegradability of the wastewater. H_2_O_2_/UV process before NF could significantly remove organic compounds and micropollutants (pesticide alachlor and hydrogen sulfide) in groundwater, thus mitigate the biofouling control and improve membrane cleanability [231]. As stated by Ganiyu et al. [226], the commonly used AOPs for pretreatment of membranes are ozonation and photolysis, with or without H_2_O_2_, while other AOPs, such as Fenton, photocatalysis and electrochemical AOPs, are not available on oxidation of low concentration pollutants in large volume water systems. AOPs as posttreatment of membrane processes are commonly used to complement membrane filtration because of its benefits of disinfection and oxidation degradation, sometimes simultaneously. Especially when applied to reclaimed wastewater on directly or indirectly potable reuse, AOPs as the terminal process can reduce micropollutants, microorganisms and trace organic contaminants that are non-rejected by membranes, under limited levels, for drinking water. Based on the reused cases of membranes combined with AOPs in Table 12, UV/H_2_O_2_ is found to be the common combination of AOPs used as posttreatment. As reported in two studies [232,233], both of them connected MF/RO processes with UV/H_2_O_2_ to treat secondary municipal wastewater for potable reuse, mainly aiming at eliminating the micropollutants, low molecular weight organic compounds, bacteria and viruses in the effluent. The results showed the hybrid MF/RO with UV/H_2_O_2_ processes not only ensure the effluent quality for potable reuse, but also contributes to the improvement by lowering, by 20%, economic cost and CO_2_ emission, than MBR or MF with UV/H_2_O_2_ process. While AOPs help to improve membrane filtration performance, membranes, in contrast, contribute to better results of AOPs. AOPs and membrane separation complement each other. As Moser et al. [234] stated, the hybrid MBR + H_2_O_2_/UV + NF process resulted in a 20% savings of both operational expenditures and capital expenditures, compared to the MBR+H_2_O_2_/UV process on the same wastewater treatment and provided good water quality for reuse. In addition, hybrid membranes with novel AOPs have occurred with favorable development potential. For example, the photocatalytic MBR is considered to be a promising method for organic matter removal from municipal wastewater [235], additionally, a hybrid UV–C + MF process in Membrane Photoreactor has resulted in high removal efficacy for various pathogen germs with contact times as low as 8 s [236].

Although there are many reports focused on the hybrid membrane–AOPs processes on wastewater treatment, the cases targeted at municipal wastewater treatment for reuse are fewer. The general disadvantages of AOPs include the relatively high operating costs and the limited large-scale applications of this very powerful technology. Although AOPs are effective in mineralizing most organic compounds, resulting in TOC decrease, in many cases they bring out harmful byproducts formation in the effluent [237]. AOPs are favorable in high removal efficiency on bacteria and viruses, but sometimes the reactivation of bacteria need to be controlled by disinfection before reuse. Therefore, another point worth mentioning is that even if the effluent is not toxic after various AOPs, it may become toxic after final disinfection (such as with chlorine) because interference from many emerging pollutants in wastewater could occur. Based on these unwanted reactions, the application of AOPs may also lead to a TDS and/or TOC increase, which should definitely be removed before reuse [238]. Therefore, the overall optimization of AOP (cost and water quality performance) needs to be improved for wastewater reuse treatment, especially for potable reuse. Most notably, NF or RO membranes, with excellent contaminant rejecting capacity, could reduce the reliance on AOPs, which would include a reduction in dosage requirements and total energy use.

### 2.6. Water Recovery and Energy Consumption of Membrane-Bases Processes for Water Reuse

The desired goal of researchers and companies on wastewater reuse treatment is to maximize economic benefits and provide optimum parameters for long term stable operation during membrane filtration. Although many articles are published in the field of municipal wastewater reuse, most of them focus on the feasibility and innovation of the processes, the hydraulic performance, the removal efficiency and optimization of the processes. Less are focused on the real productivity, wastewater recovery rate, energy consumption or total cost during long term operations. Among all the references mentioned in the above tables of this review, only a few of them stated the relevant energy consumption, mostly estimated in pilot scales (Table 13). The water recovery rate from wastewater by membrane-based technologies may be influenced by feed water characteristics, the permeate consumption by physical and chemical cleaning and filtration time, etc. The net energy consumption may depend on the treatment technologies, the chemical reagents consumption and other supplementary energy production (e.g., methane), etc.

From Table 13, low-pressure driven membrane technologies (MF, UF, MBR) support higher productivity per unit area and present higher water recovery, with over 85% recovery rate. The water recovery for high-pressure driven membranes (NF, RO) and FO are relatively lower. Generally, the water recovery by RO itself (the percentage of permeate separated from the feed water) ranges from 50–75% because the remaining 25–50% of the RO feed water is usually concentrated with waste [247]. Some studies have reprocessed the RO concentrate for reuse. For example, the hybrid process of RO- electrodialysis reversal by Venzke et al. [248] improved the water recovery from 75% to 87.3%, but the applied technology is complex with high energy consumption. In addition, although FO is advantageous considering the operating pressures leading to low energy consumption, the wastewater recovery rate is not high. As studied by Singh et al. [249], the water recovery by FO was only 57% with an average flux of 5.3 L·m^−2^·h^−1^ over 24 h’s operation.

In net energy comparison, low-pressure driven membrane technologies always have lower energy consumption. According to Atanasova et al. [149], with an inlet flowrate to an MBR over 15 m^3^·d^−1^, the net energy consumption was less than 0.5 kWh m^−3^, even including the cost of disinfection. The net energy costs of an MBR on municipal wastewater treatment are relatively low ranging from 0.19 to 1.07 kWh·m^−3^ [137,250]. It should be noted the above data can only be used as a reference, not as the actual energy consumption for municipal wastewater reuse treatment. In addition, the processes with NF, RO and FO directly increased the energy consumption without consideration of biogas for energy recovery (Table 13), because of the increased TMP, membrane maintenance, etc. The hybrid MBR +RO processes in Table 13 have net energy consumption around 0.518 to 0.739 kWh·m^−3^ [145,184], while the combined FO+NF system consumed 3.44–4.57 kWh·m^−3^, which is not suitable for industrial scale application because of high economic cost. However, it is worth discussing the energy recovery by biogas generated in AnMBR. With the energy recovery by methane, the AnMBR + RO system could save 0.185 kWh·m^−3^ net energy and the AnMBR + RO + IE system could save 0.547 kWh·m^−3^ net energy [145,185].

In conclusion, as seen in real cases of municipal wastewater reuse treatment, the wastewater recovery rate and net energy consumption are more likely considered as the two main factors, on the basis of meeting required reuse level, because they are important indicators to determine whether the method is cost effective or not. In the future, it could be more interesting to focus on the economic prediction of membraned-based technologies, which will be the reference for the future development of wastewater reuse field.

## 3. Challenges, Prospects and Conclusions

While the reuse of municipal wastewater presents a promising approach to reduce water pollution and release water scarcity, the proportion of reused wastewater is quite low. The total volume of water reuse is approximately 14.2 billion m^3^·y^−1^ worldwide reported in Global Water Market 2017, which is less than 4% of the total volume of domestic wastewater (250 to 350 billion m^3^·y^−1^) [6,26]. Actually, the reuse proportion differs significantly among various countries and regions based on real conditions. In extreme water shortage areas, the Arab Region, 23% of collected wastewater is being reused, mostly for irrigation and groundwater recharge [10]. The Singapore government, with the will to be independent from water demand, reaches 40% reuse of total wastewater. Some countries like Israel and Tunisia keep ahead in wastewater reuse-with-reuse rates over 80%. In addition, the USA as a pioneer in waster reuse concepts and technology holds over one-fifth of the reused water capacity worldwide whereas it only provides a national water reuse rate of 9.7%. In Europe, approximately 2.4% of the total treated urban wastewater effluent is reused. Under this global situation, the water reuse approach is a promising market with high potential. As the European Parliament estimated with its proposal in May, 2020, increasing water reuse in agricultural irrigation from 1.7 billion m^3^·y^−1^ to 6.6 billion m^3^·y^−1^ by 2025 could reduce water stress by 5% [37]. Generally, the implementation of wastewater facilities requires a substantial capital expense. Under the integrated water resources management of a region, the implementation of wastewater reuse may require government grants or subsidies. Unfortunately, institutional barriers, financial allocation, and different agency priorities can make water reuse projects difficult to progress in some cases.

Currently, reused water production from various membrane-based treatment fulfils multiple water use objectives. However, the ultimate decision to promote wastewater reuse depends on economic factors, government regulations and policies, and most importantly, public acceptance reflecting on the water demand, security and requirements for reliable water supply in local conditions. The wastewater reuse guidelines are not unified in the world even for the same water reuse purpose. The “Guidelines for the safe use of wastewater, excreta and greywater in agriculture and aquaculture”, published by the WHO in 2006, is considered as the most relevant document regulating wastewater reuse, being widely adopted or referred to, by many countries [251]. Moreover, some countries develop stronger reuse standards that provide more guarantees of safe reuse. For example, the reuse standard in France supplements the regulation of microorganisms, including Fecal Enterococci, F-specific phages FNA and Spore of sulfating anaerobic bacteria, than the WHO [252]. However, upon investigation by M. Gurel et al. [253], it is seen that in the U.S., quality limits differ from state-to-state, and that implementing a single standard seems difficult due to national and/or local circumstances. In parallel to scientific evidence and advanced technologies, continuous improvement in the existing reuse standards and guidelines by revising/expanding is necessary. In addition, control and monitoring during reuse applications along with the public feedback is necessary for safety assessment. It is also worth noting that cultural and religious differences direct public opinion and acceptance of wastewater reuse. In addition, some developing countries still implement the direct use of treated wastewater without accordance with standards and associated risks. In this case, it is suggested to enact wastewater quality standards step-by-step, over suitable periods and according to treatment capabilities, for developing countries.

Anti-fouling is always the research hotspot in membrane filtration. In this case, a periodical cleaning procedure is necessary for each membrane filtration system. In addition, many studies are focused on the membranes anti-fouling enhancement by selection of new starting monomers, improvement of interfacial polymerization process, the hybrid organic/inorganic membrane, and more attractively, the surface modification of conventional membranes by physical and chemical methods [254,255,256]. In general, the modified membrane permeation performance can be improved significantly without sacrificing its retention properties, and its anti-fouling performance is more favorable than the unmodified one [257,258]. Thus, the modified membrane is a promising option for treating produced water, especially if applied in the wastewater reuse field or compliance with disposal regulations.

The raw wastewater quality variation in terms of living styles, seasonal change and peak periods, could finally influence the treatment performance, such as the effluent quality, organic load rates and fouling mechanisms [121,259,260]. Moreover, although many studies achieved reuse objectives successfully as mentioned in the last sections, the published reports still lack long-term and/or large-scale (industrial scale) operation and monitoring of the filtration performance and optimization of fouling control strategies. In these cases, studies should focus much closer on real conditions of the targeted wastewater, make comprehensive consideration of the potential impact factors as much as possible, and deliberate optimization of operational conditions under non-ideal situations.

During long-term investigation, it can be logically predicted that successful non potable reuse is more likely to build the degree of familiarity and trust required by the public for acceptance [261]. In the global water reuse market, 97.7% of water production is reused for non-potable purposes, of which 52% is for irrigation and 20% is for industrial process water recirculation. On the other hand, only 2.3% of water production is used for potable purposes and mostly for indirect potable reuse [262]. For instance, the NEWater in Singapore’s potable reuse application cannot be directly used, instead, it needs to be discharged into reservoirs to mix with rainwater before being collectively treated in the water treatment plants for potable use [22]. The reason why the distribution of potable reuse is low (2.3%) could mainly be blamed on public concerns of health risks and expensive cost of advanced treatment processes. However, highly treated water production, when it is successfully used in more practical applications (e.g., potable water), may earn enough support to make up for the high cost of treatment. In the process of collecting references, we found more published research articles focusing on NF/RO processes aimed at achieving acceptable potable reuse levels, compared to the MF/UF/MBR based treatment processes. With the advanced science development, driven by increasing demand for high quality potable water, population growth and limited resources, it is expected we’ll see an expansion in the potable reuse applications, especially in water scarcity regions in the future [263]. This requires ensured access to credible scientific information for the public, decision makers and the local media, toward improving understanding of water supplies in the region and the costs/benefits analysis of water supply treatment options.

Lastly, several studies performed life cycle assessment of wastewater reclamation as a decentralized approach to urban wastewater management [264,265,266]. They emphasized that local wastewater reclamation is environmentally preferable to the common centralized system, especially for non-potable uses, agricultural and urban, with environmental and economic advantages. Reuse of treated wastewater is particularly beneficial when it can replace desalinated water [264]. Therefore, a comprehensive life cycle assessment of membrane-based techniques on municipal wastewater treatment is critically needed, especially in different scenarios (developing countries vs. developed countries; countries facing water scarcity vs. countries with sufficient water; countries with electricity as energy source vs. countries with sufficient renewable energy).

This review gave a comprehensive elaboration on the global water reuse situation, reuse regulation and membraned-based treatment performance on municipal wastewater reuse. The membrane-based treatment processes, such as direct membrane filtration and hybrid membrane systems, have been mainly evaluated in this review. The advantages and limitations of them on reuse purposes, are listed in Table 14. Membrane separation processes often provide superior quality to the treated wastewater to meet local reuse guidelines. This work emphasized the performances of the different treatment processes: the pollutant removal capacity, microorganism removal, membrane fouling potential, productivity, reuse purposes, water recovery rate and energy consumption. Furthermore, future research focusing on micropollutant removal, fouling mechanism strategies, resource recovery, and life cycle assessment of membrane-based municipal treatment strategies, for potable and non-potable reuse applications, continues to need further systematic investigation.

## Figures and Tables

**Table 1:** Major water reuse applications and constraints worldwide [7,8].

Application		Major Constraints	Percentage Contribution
Types	Examples		
Potable reuse	Indirect and direct drinking	Public perception issues	2.3%
Non potable urban reuse	Public parks and schoolyards Highway medians Residential landscapes Fire protection Toilet flushing	Dual distribution system costs The requirement for dual piping systems The greater burden on cross connection control	8.3%
Agricultural Irrigation	Nonfood crops Commercial nurseries Pasture lands	Seasonal demand Usually away from the point of water reclamation Public perception issues High–total dissolved solids (TDS) reclaimed water can adversely affect plant health	32%
Landscape Irrigation	Parks and schoolyards Roadway medians Residential lawns Golf courses Cemeteries Greenbelts	Seasonal demand Usually away from the point of water reclamation High TDS reclaimed water can adversely affect plant health	20%
Recreation	Ponds and lakes Golf courses	Site specific	6.4%
Environmental enhancements	Artificial wetlands Natural wetlands Stream flows	Site specific	8%
Industries	Process water Boiler water makeup Cooling tower water Geothermal energy	Constant demand, but site-specific Limited demand Treatment required depends on end-use	19.3%
Groundwater recharge	Groundwater replenishment Barrier against brackish or seawater intrusion Ground subsidence control	Appropriate hydrogeological conditions needed High level of treatment required Potential for water quality degradation in the subsurface	2.0%
Others			1.7%

**Table 2:** Water reuse capacity in typical regions from 2006 to 2017 [6,11].

Country/Region	Water Reuse Volume Estimation (×10^6^ m^3^·d^−1^)	Water Reuse Rate *	Year	Reference
U.S.A	13.0	9.7%	2000	[12]
China	5.90	2.9%	2015	[13]
Arab region (Arabian Peninsula)	3.63	23%	2013	[10]
India	3.54	30%	2017	[14]
Europe	2.65	2.4%	2006	[15]
Korea	2.58	13.5%	2014	[16]
Israel	1.1	87%	2016	[17]
Australia	0.8	16.8%	2010	[18]
Mexico	0.67	9.0%	2010	[19]
Japan	0.59	1.5%	2014	[20,21]
Singapore	0.58	40%	2013	[22]
Tunisia	0.4	83%	2006	[23,24]

* water reuse rate is the ratio of reused water volume over the total wastewater collected.

**Table 3:** Constituents present in wastewater with their associated risks [28,33,34,35].

Wastewater Constituents	Contents	Risks
Microorganisms	Pathogenic bacteria, viruses and worms’ eggs	Risk when exposed to humans and animal by inhalation or drinking
Micropollutants	Pesticides, pharmaceuticals, fuel additives, cyanotoxins, personal care products, detergents	Environmental as well as further expected impacts on humans, such as genotoxic, immunotoxic, carcinogenic and fertility-impairing effects
Suspended solids	Particles, solids, colloids	Carrying pollutants and pathogens
Biodegradable organic matters	Organic carbon, sugar, protein, ammonia	Oxygen depletion in rivers, lakes and fjords; fish death; odors
Other organic matters	Fat, oil and grease, coloring, solvents, phenols	Toxic effect, esthetic inconveniences, bioaccumulation in the food chain
Other Nutrients	Nitrogen (ammonium, nitrates), phosphorus	Eutrophication, oxygen depletion, toxic effect
Metals	Hg, Pb, Cd, Cr, Cu, Ni	Toxic effect, bioaccumulation
Others inorganic materials	Acids, for example, hydrogen sulfide, bases	Corrosion, toxic effect
Thermal effects	Hot water	Changing living conditions for flora and fauna
Odor (and taste)	Hydrogen sulfide	Esthetic inconveniences, toxic effect
Radioactivity		Toxic effect, accumulation

**Table 4:** Water reuse categories and typical application (WHO guidelines and EU Parliament regulation project).

Organization	Category	Typical Application	pH	TSS (mg·L^−1^)	Turbidity (NTU)	BOD_5_ (mg·L^−1^)	Residual Cl^−1^ (mg·L^−1^)	Fecal Coliform (*E. coli* as an Indicator) (100 mL^−1^)
EU Parliament	Agriculture irrigation	A		≤10	≤5	≤5		≤ 10/below detection limit
		B		Directive 91/271/EEC	–	Council Directive 91/271/EEC		≤100
		C			–			≤1000
		D			–			≤10,000
WHO guidelines	Agriculture irrigation	Food crop irrigation (uncooked)	6–9	ND	≤2	≤10	1	ND
		Non-food crops and crops consumed after processing	6–9	≤30	–	≤30	1	≤ 200
	Landscape irrigation	Parks; schoolyards; Playgrounds	6–9	ND	≤2	≤10	1	ND
		Golf courses; Cemeteries; Greenbelts; Residential	6–9	≤30	–	≤30	1	≤200
	Industrial recycling and reuse	Cooling water; boiler feed; Process water; Heavy construction	–	≤30	–	≤30	–	≤200
	Groundwater	Groundwater replenishment; saltwater intrusion control; Subsidence control	Site-specific; specific guidelines do not exist.					
	Recreational Environmental uses	Lakes and ponds; marsh enhancement; streamflow augmentation; fisheries; snowmaking	6–9	ND	≤2	≤10	1	ND
	Non-potable urban uses	Fire protection; air conditioning; toilet flushing	6–9	ND	≤2	≤10	1	ND
	Potable uses	Blending in water supply reservoirs; Blending in groundwater; Direct pipe-to-pipe water supply	Meet requirements for safe drinking water; specific guidelines do not exist.					

ND, not detected; NTU, nephelometric turbidity units; ABCD represents the different water quality levels, A being the best, D being the worst.

**Table 5:** Membrane separation characters for water purification [52,63,64,65,66].

Characters	MF	UF	NF	RO (Low-Pressure)
Separation mechanism	Sieve	Sieve	Sieve, solution/diffusion, Exclusion, electric repulsion	Solution/diffusion, Exclusion
Membrane	Porous isotropic	Porous asymmetric	Finely porous asymmetric/composite	Nonporous asymmetric/composite
Molecular weight cutoff	>1000 kDa	1–300 kDa	200–1000 Da	–
Retained compounds	Colloids, TSS turbidity, some protozoan oocysts, cysts, some bacteria and viruses	Macromolecules, proteins, colloids, bacteria, viruses	LMWC, mono-, di- and oligo-, saccharides; polyvalent anions, some hardness, viruses	LMWC, sodium, chloride, glucose, amino acids, hardness, ions
Transmembrane pressure (TMP)	<5 bar	<10 bar	<20 bar	<100 bar
Flow modes	Crossflow, Dead-end	Crossflow, Dead-end	Crossflow	Crossflow
Geometry	Hollow fiber, spiral wound, plate and frame, tubular	Hollow fiber, spiral wound, plate and frame, tubular	Hollow fiber, spiral wound, tubular	Hollow fiber, spiral wound

HMWC: high molecular weight compounds; LMWC: low molecular weight compounds.

**Table 6:** Microfiltration (MF)/ultrafiltration (UF) processes alone after secondary treatment for water reuse applications.

Process (scale and Operating Duration)	Feed Wastewater	Operating Conditions	Feed Characteristics	Permeate Quality/Removal Rate	Application for Reuse	Standard Basis	References
MF (pilot plant, 120 d)	Domestic wastewater (septic tank effluent)	Capacity: 10 m^3^·d^−1^ MF: 0.1 μm; TMP: 0.20–0.50 bar	COD: 10–622 mg·L^−1^ BOD_5_: 25–110 mg·L^−1^ TOC: 2.8–22.6 mg·L^−1^ TSS: 5–645 mg·L^−1^; Turbidity: 2.7–123 NTU Color: 5–109 CU	COD: 1–30 mg·L^−1^, 92.8% BOD_5_: 1–7 mg·L^−1^, 92.9% TOC: 0.4–8.1 mg·L^−1^, 65.8% TSS: 0–2 mg·L^−1^, 99.8% Turbidity: 0–4.2 NTU, 99.4% Color: 2–32 CU, 76.2%	Toilet flushing	Not mentioned	[75]
UF (pilot plant, 2 years)	Secondary effluent of WWTP	TMP: 0.1–0.7 bar UF: 0.03 μm	TSS: 96–165 mg·L^−1^ COD: 167–307 mg·L^−1^ PO_4_–P: 1.0–3.9 mg·L^−1^ NH_4_–N: 3.0–33 mg·L^−1^ N*_org_*: 9–16 mg·L^−1^	TSS: 3–9 mg·L^−1^ COD: 42–103 mg·L^−1^ PO_4_–P: 0.8–3.4 mg·L^−1^ NH_4_–N: 4.0–33 mg·L^−1^ N*_org_*: 2–5 mg·L^−1^ LRV (total coliforms): 3.7 LRV (fecal coliforms): 4.2 LRV (*E. coli*): 3.7	Crops irrigation (tomato and fennel)	Meeting WHO guidelines	[81]
Prefilter +UF(pilot plant, two months)	Secondary effluent of WWTP	UF: 0.01 μm (200 kDa), UF modes: crow flow Inlet flow: 10 m^3^·h^−1^ TMP: 0.30–1.20 bar	pH: 6.3–7.5 T: 19–25 °C EC: 1584–1950 μS·cm^−1^ Turbidity: 1–7 NTU TSS: 1–8 mg·L^−1^ COD: 26–69 mg·L^−1^ *E. coli*: 3000–36,000 CFU·100 mL^−1^ Total coliforms: 9100–96,000 CFU·100 mL^−1^	Turbidity: < 0.2 NTU TSS: < 0.2 mg·L^−1^ COD: 20–60 mg·L^−1^ *E. coli*: 0 CFU·100 mL^−1^ Total coliforms: 0 CFU·100 mL^−1^	Agriculture irrigation	Meeting WHO guidelines	[77]
Prefilters +UF (pilot scale)	Synthetic secondary sewage effluent	UF: 1 kDa/0.002 μm (tubular); 25 kDa/0.008 μm (spiral wound) UF modes: cross flow TMP: 1.0–3.3 bar Flow velocity: 0.2 m·s^−1^	COD: 18.5–67 mg·L^−1^ Turbidity: 9.43–46.4 NTU TSS: 13–30 mg·L^−1^ Color: 41–81 EC: 320–366 μS·cm^−1^ A(254 nm): 0.25–1.051 pH: 7.7–7.81	COD: 64.38–80.4% Turbidity: 96.75–99.61% Color: 0–53.49% Absorbance (254 nm): 76.6–91.94%	Non-potable reuse (not detailed)	Meeting WHO guidelines	[78]

A: absorbance; CFU: colony forming unit; EC: electrical conductivity; LRV: log10 removal value; SAC: spectral absorption coefficient.

**Table 7:** Hybrid MF/UF membrane with chemical/physical processes after conventional secondary treatment for water reuse applications.

Membrane Process (Scale and Operation Duration)	Feed Wastewater	Operating Conditions	Feed Characteristics	Permeate Quality/Removal Rate	Application for Reuse	Standard Basis	References
GAC+MF (submerged) (lab scale,140 d)	Secondary treated water of the sewage treatment plant in Sung- kyunkwan University	MF: 0.22 μm Flux: 98 L·m^−2^·h^−1^ TMP: 0–0.4 bar	pH: 7.62–8.02 Turbidity: 2.2–10.3 NTU TSS: 4–20 mg·L^−1^ UV_260_: 0.28–0.32 cm^−1^ DOC: 6–8 mg·L^−1^ COD: 10–30 mg·L^−1^ TN: 30–50 mg·L^−1^ TP: 15–30 mg·L^−1^	Turbidity: 0.1–0.4 NTU, 100% TSS: 100% UV_260_: 0.26–0.3 cm^−1^, 60% DOC: 2–4 mg·L^−1^, 40–46% COD: 8–25 mg·L^−1^, 53% TN: 20–40 mg·L^−1^ 15% TP: 10–20 mg·L^−1^, 13%	Not mentioned	WHO guidelines	[100]
Submerged MF–GAC (Semi-batch, 60 d)	Biologically treated sewage effluent	MF: 0.14 μm, flat sheet NF: 700 Da, flat sheet TMP: ≤ 4 bar Flux: 2.5 L·m^−2^·h^−1^ GAC: 10% daily replacement	pH: 6.8–7.6 EC: 520–1120 μS·cm^−1^ DOC: 3.6–7.7 mg·L^−1^	DOC: 2.4 ± 0.2 mg·L^−1^, 53 ± 5% PPCPs: < 5 ng·L^−1^ (for each)	Not mentioned	Australian and New Zealand Guidelines for Fresh and Marine Water Quality	[88]
Coagulation +MF (lab scale, 5 months)	Secondary effluent from WWTP	Coagulant: 10–50 mg·L^−1^ alumina MF: 0.1, 0.22 μm; TMP: 0.34 bar	Turbidity: 19.7 ± 87.9 NTU TOC: 7.2 ± 6.5 mg·L^−1^ pH: 7.0 ± 0.2 UV_254_: 0.040–0.058 cm^−1^ Alkalinity: 202.8 ± 12.2 mg·L^−1^ as CaCO_3_ TSS: 14.4 ± 25.8 mg·L^−1^	Turbidity: 0.11–0.13 NTU, >93%; TOC: 1.30–1.56 mg·L^−1^, 23.5–35.5% UV_254_: 0.019–0.02 cm^−1^, 52.5–54.5%	Not mentioned	Not mentioned	[99]
Coagulation + PAC + UF (lab scale)	Secondary effluent from WWTP	UF: 50 kDa TMP: 1 bar Coagulant: FeCl_3_	pH: 7.4 Turbidity: 18 NTU TSS: 35 mg·L^−1^ BOD_5_: 30 mg·L^−1^ COD: 77 mg·L^−1^ EC: 1350 μS·cm^−1^ Zeta potential: 4.118 mV	COD: 13.33–21 mg·L^−1^ Turbidity: 0.5–0.8 NTU Zeta potential: −0.332–0.166 mV	Not mentioned	Not mentioned	[106]
PAC+UF (lab scale, ~1 h)	Secondary effluent from domestic WWTP	UF: 100 kDa TMP: 1 bar	pH: 7.1–7.6 Turbidity: 0.9–1.5 NTU TOC: 3.3–5.2 mg·L^−1^ UV_254_: 0.09–0.12 cm^−1^ COD: 25–32 mg·L^−1^ Color: 18–24 CU Coliforms: 300–700 mL^−1^	DOC: 22.2–28.8% UV_254_: 33.7–38.3%	urban reuse, agricultural, landscape and industrial reuse	Not mentioned	[107]
MF+UF	Secondary effluent from urban WWTP	MF: 0.2 μm, hollow fiber, 0.2–0.8 bar (TMP) UF: 0.05 μm, flat sheet, 0.2–0.6 bar (TMP)	Turbidity: 4–20 NTU TSS: 11–87 mg·L^−1^ T-UV_253.7_: 11–41% Nematode eggs: 0–200 Un·L^−1^ *E. coli*: 10^4^–10^6^ CFU·100 mL^−1^ Fecal coliforms: 10^4^–10^6^ CFU·100 mL^−1^ Coliphages: 10^3^–10^4^ PFU·100 mL^−1^	Turbidity: 0–0.9 NTU TSS: 1–7 mg·L^−1^ T-UV_253.7_(%): 0 Nematode eggs: 0 *E. coli*: 0 CFU·100 mL^−1^ Fecal coliforms: 0–9 CFU·100 mL^−1^ Coliphages: 0–1 PFU·100 mL^−1^	Not mentioned	Water reuse guidelines of US EPA	[108,109]

CFU: colony forming unit; EC: electrical conductivity; PPCPs: pharmaceuticals and personal care products; SAC: spectral absorption coefficient.

**Table 8:** Membrane bioreactors (MBR) for water reuse applications.

Membrane Process (Scale and Operating Duration)	Operating Conditions	Feed Characteristics	Permeate Quality/Removal Rate (in Average)	Application for Reuse	Standard Basis	References
Aerobic MBR (pilot plant, 30 d)	Feed: mixed municipal and industrial wastewater MF: 0.4 μm, flat-sheet Flux: 83 L·m^−2^·h^−1^ MLSS: 1600–2300 mg·L^−1^ HRT: 8 h SRT: 25 days	pH: 7.3 ± 0.62 SS: 223 ± 32 mg·L^−1^ COD: 250 ± 64 mg·L^−1^ Al: 250 ± 70 µg·L^−1^ Fe: 180 ± 80 µg·L^−1^ Pb: 340 ± 190 µg·L^−1^ Cu: 610 ± 170 µg·L^−1^ Ni: 160 ± 90 µg·L^−1^ Cr: 225 ± 105 µg·L^−1^ Coliforms: 10^6^ MPN·100 mL^−1^	SS: <5 mg·L^−1^, >98% COD: 41–51 mg·L^−1^, >75% Al: 81% Fe: 53% Pb: 94% Cu: 91% Ni: 59% Cr: 49% Coliforms: < 140 MPN·100 mL^−1^, 99.9%	Reused for process water in industries, cleaning, recreational water supplies or discharged to surface waters.	Not mentioned	[148]
Aerobic MBR+ GAC (water recycle plant, 12 months)	Feed: Primary effluent of municipal wastewater recycling plant UF: 0.04 μm HRT: 3.2 h (MBR) + 0.58 h (GAC)	BOD5: 46.2–262.1 mg·L^−1^ COD: 142.0–512.0 mg·L^−1^ SS: 47.5–240 mg·L^−1^	BOD5: < 1.9 mg·L^−1^, >96% COD:< 48.3 mg·L^−1^, >65.9% SS: < 7.2 mg·L^−1^, >85% Fecal coliforms: 0.3 CFU·100 mL^−1^ Phages: 3.9–5.6 log reduction	Non-potable	California Department of Public Health	[116]
Aerobic submerged MBR (pilot-scale, 50 days)	Feed: black water from household MF: 0.4 μm Flux: 30–40 L·m^−2^·h^−1^ HRT: 36 h	pH: 7.6 BOD_5_: 406 mg·L^−1^ Total COD: 1218 mg·L^−1^ Soluble COD: 417 mg·L^−1^ TN: 188 mg·L^−1^ NH_4_^+^-N: 155 mg·L^−1^ TP: 21.3 mg·L^−1^ TSS: 560 mg·L^−1^ Total coliform: >10^6^ 100 mL^−1^	BOD_5_: 8 ± 4 mg·L^−1^, 98% Total COD: 42 ± 8.81 mg·L^−1^, 96% TSS: 2 ± 1.19 mg·L^−1^, 99% TN: 19 ± 4.73 mg·L^−1^, 89% NH_4_^+^-N: 11 ± 3.76 mg·L^−1^, 92% NO_X_-N: 8 ± 3.1 mg·L^−1^ Total coliforms: 0, 100%	Toilet flushing, Cleaning, Irrigation	Water reuse standards of EPA, EU, WHO, Turkey	[114]
Aerobic submerged MBR (pilot plant, 6 months)	Feed: hotel greywater UF: 0.04 μm, hollow fiber Flux: 20 L·m^−2^·h^−1^	COD: 41–500 mg·L^−1^ BOD_5_: 36–295 mg·L^−1^ TN: 2.6–25 mg·L^−1^ Ammonia N: 0.3–14 mg·L^−1^ TP: 0–6.7 mg·L^−1^ Total count: 1.5 × 10^7^–4.1 × 10^7^ CFU·100 mL^−1^ Total coliforms: 1.4 × 10^6^–4.1 × 10^6^ CFU·100 mL^−1^ *E. coli*: < 1.1 × 10^6^ CFU·100 mL^−1^	COD: <36 mg·L^−1^ TN:<10 mg·L^−1^ Ammonia N: <8 mg·L^−1^ Total count: 5.8 × 10^3^–1.6 × 10^5^ CFU·100 mL^−1^ Total coliforms: 0.27 × 10^2^–2.1×10^2^ CFU·100 mL^−1^ *E. coli*: <1.1 × 10^2^ CFU·100 mL^−1^ Intestinal Enterococci /Nematodes: < 1 CFU·100 mL^−1^ *Legionella spp.*: < 1 CFU·100 mL^−1^	Non-potable reuse	Spanish water reuse standard	[149]
Aerobic MBR (External): Pilot plant for 162 days	Feed: urban wastewater UF: 0.02 μm/300 kDa Flux: 75–150 L·m^−2^·h^−1^ HRT: 5 d SRT: 5–30 days	COD: 200–800 mg·L^−1^ SS: 100–600 mg·L^−1^ NH_3_-N: 10–30 mg·L^−1^ Coliform: 10^5^–10^6^·L^−1^ Turbidity: 50–70 NTU pH: 7.5–8.5	COD: 9.4 mg·L^−1^, 97%; SS: nd,100%; NH_3_-N: 0.2–1.3 mg·L^−1^, 96.2%; Turbidity: < 2 NTU Coliform: nd pH: 8.2	Directly for municipal purposes or indirectly for industrial uses after additional treatment	Water reuse standard of China	[117]
Vertical submerged MBR (pilot scale for 600 days)	Feed: municipal wastewater in Korea UF: 0.45 μm Flux: 6.2 L·m^−2^·h^−1^ HRT: 8 h SRT: 60 days	COD: 232 ± 41 mg·L^−1^ TSS: 220 ± 52 mg·L^−1^ TN: 42 ± 5 mg·L^−1^ TP: 3.2 ± 0.4 mg·L^−1^ Volatile fatty acids: <1.0 mg·L^−^^1^ pH: 7.3 ± 0.1 Alkalinity as CaCO_3_: 145 ± 47 mg·L^−^^1^	COD: 9.0 ± 3.6 mg·L^−1^, 96% TSS: 220 ± 52 mg·L^−1^ TN: 10.6 ± 2.6 mg·L^−1^, 74% TP: 0.7 ± 0.2 mg·L^−1^, 78% Total colony counts: 24 CFU·mL^−1^ Turbidity: 0.18 NTU, pH: 7.3	Urban or rural reuse, such as toilet flushing, sprinkling and car washing	Drinking water standards of Korea and the WHO	[150]

HRT: hydraulic retention time; SRT: solids retention time; nd: not detected.

**Table 9:** Nanofiltration (NF)/reverse osmosis (RO)/ forward osmosis (FO) coupled with pretreatment processes for wastewater reuse applications.

Process (Scale and Operation Duration)	Feed Water	Operating Conditions	Feed Characteristics	Final permeate quality (Removal rate)	Application for Reuse	Standard Basis	References
(Biologic methods) + NF (lab scale, 6 h)	Bio-treated municipal wastewater	TMP: 10 bar NF: 150 Da (CA), 200 Da (PTFC), flat sheet	TDS: 3150–3908 mg·L^−1^ EC: 6303–7815 μS·cm^−1^ pH: 8.13–8.34 Salinity: 3.51–4.17 g·kg^−1^ Turbidity: 0.15–0.39 NTU Na^+^: 1018–1091 mg·L^−1^ Ca^2+^: 195–218 mg·L^−1^ K^+^: 80.3–96.8 mg·L^−1^ Mg^2+^: 134–150 mg·L^−1^ NH_4_–N: 0.10–0.11 mg·L^−1^ HCO_3_^−^: 408–440 mg·L^−1^ SO_4_^2−^: 261–299 mg·L^−1^ Cl^−^: 1834–1848 mg·L^−1^ PO_4_–P: 2.10–2.28 mg·L^−1^ NO_3_–N: 8.55–8.65 mg·L^−1^ NO_2_–N: 0.21–0.22 mg·L^−1^ Si: 10.5–12.5 mg·L^−1^ COD: 29.8–30.5 mg·L^−1^ Color: 21.1–22.3 Hazen TOC: 13.7–16.5 mg·L^−1^	TDS: 340–1150 mg·L^−1^ EC: 690–2300 μS·cm^−1^ pH: 7.59–7.60 Salinity: 0.34–1.16 g·kg^−1^ Turbidity: <0.02 NTU Na^+^: 134–353 mg·L^−1^ Ca^2+^: 0.54–7.12 mg·L^−1^ K^+^: 14.5–30.7 mg·L^−1^ Mg^2+^: 0.41–4.8 mg·L^−1^ NH_4_–N: 0.06–0.07 mg·L^−1^ HCO_3_^−^: 31–57.2 mg·L^−1^ SO_4_^2−^: <0.05 mg·L^−1^ Cl^−^: 206–572 mg·L^−1^ PO_4_–P: <0.05 mg·L^−1^ NO_3_–N: 3.75–5.63 mg·L^−1^ NO_2_–N: 0.06–0.07 mg·L^−1^ Si: 4.07–4.75 mg·L^−1^ COD: 4.49–7.18 mg·L^−1^ Color: 1.85–2.1 Hazen TOC: 1.7–3.34 mg·L^−1^	Irrigation	FAO Irrigation and Drainage Paper	[160]
Submerged MBR+ NF	Municipal wastewater after primary treatment	UF: 200 kDa, hollow fiber NF: 150–300 Da, TMP: 0.1–0.5 bar (UF); 41 bar (NF)	(raw wastewater) EC: 1174 ± 2 μS·cm^−1^ pH: 7.22 ± 0.11 TSS: 488 ± 48 mg·L^−1^ Turbidity: 248 ± 11 NTU DOC: 126.6 ± 7.3 mg·L^−1^ COD: 478 ± 132 mg·L^−1^ F^−^: 0.096 ± 0.003 mg·L^−1^ Cl^−^: 156.0 ± 2.4 mg·L^−1^ NO_2_^−^: 64.35 mg·L^−1^ NO_3_^−^: 144.53 ± 42.17 mg·L^−1^ PO_4_^3−^: 9.631 ± 1.428 mg·L^−1^ SO_4_^2−^: 36.33 ± 0.84 mg·L^−1^ Na^+^: 71.14 ± 0.48 mg·L^−1^ K^+^: 11.85 ± 0.14 mg·L^−1^ Mg^2+^: 22.05 ± 0.04 mg·L^−1^ Ca^2+^: 110.7 ± 0.2 mg·L^−1^ SAR: 1.61 ± 0.01	EC: 397 μS·cm^−1^ pH: 8.06 TSS: 0 mg·L^−1^ Turbidity: 0.23 NTU DOC: 0.35 mg·L^−1^ COD: < 5 mg·L^−1^F^−^: n.a. mg·L^−1^ Cl^−^: 63.77 mg·L^−1^ NO_2_^−^: 0.3728 mg·L^−1^ NO_3_^−^: 63.1 mg·L^−1^ PO_4_^3−^: n.a. mg·L^−1^ SO_4_^2−^: 0.464 mg·L^−1^ Li^+^: nd Na^+^: 38.01 mg·L^−1^ NH_4_^+^: nd K^+^: 5.9 mg·L^−1^ Mg^2+^: 3.04 mg·L^−-1^ Ca^2+^: 29.9 mg·L^−1^ SAR: 1.77	Agricultural Irrigation (50% of MBR effluent and 50% of NF permeate)	WHO and FAO guidelines	[159]
NF	MBR effluent from domestic wastewater	Lp_0_: 6.2 L·m^−2^·h^−1^·bar^−1^ TMP: 8 bar	UV_254 nm_: 0.148–0.155 UV_210 nm_: 1.579–3.207 TOC: 6.0–8.0 mg·L^−1^ COD: 12–13 mg·L^−1^ TSS: <2 mg·L^−1^ Mg^2+^: 8.9–9.8 mg·L^−1^ Ca^2+^: 25.0–28.4 mg·L^−1^ EC: 631–894 μS·cm^−1^	UV_254 nm_ > 95% UV_210 nm_: 75–81% TOC: 82–95% EC: 92–94%	Not mentioned	Not mentioned	[166]
UF+NF	Synthetic municipal wastewater after aerobic activated sludge process	UF: 30 kDa, PES NF: 270 Da, PA TMP: 1–6 bar	COD: 243.34 mg·L^−1^ TP: 7.53 mg·L^−1^ NH_3_–N: 0.67 mg·L^−1^ NO_2_-N: 4.32 mg·L^−1^ NO_3_–N: 34.43 mg·L^−1^	COD: 3.68 mg·L^−1^ TP: 0.19 mg·L^−1^ NH_3_–N: 0.14 mg·L^−1^ NO_2_-N: 0.14 mg·L^−1^ NO_3_–N: 1.37 mg·L^−1^	Inner industrial reuse, Garden irrigation	China municipal water reuse standards	[206]
Forward osmosis (FO) + NF	Secondary effluent from WWTP	Flux: 2.4 L·m^−2^·h^−1^ for FO Flux: 3.3 or 6.6 L·m^−2^·h^−1^ for NF	*E. coli*: 0 CFU·100 mL^−1^ TSS: < 1 mg·L^−1^ Turbidity: 0.22 NTU EC: 5.33 dS·m^−1^ SAR: 10.6 meq·L^−1^ B: 1.17 mg·L^−1^ Arsenic: 0.0015 mg·L^−1^ Chrome: 0.0041 mg·L^−1^ Copper: 0.002 mg·L^−1^ Manganese: 0.018 mg·L^−1^ Molybdenum: 0.002 mg·L^−1^ Nickel: 0.0016 mg·L^−1^ Selenium: <0.004 mg·L^−1^	Conductivity: 1 mS·cm^−1^ B: < 0.4 mg·L^−1^ SAR: 1.98 meq·L^−1^	Agricultural irrigation	Spanish water reuse legislation (RD1620/2007)	[197]
OMBR + RO	Synthetic wastewater	FO: flat sheet, thin-film composite (TFC), 0.5-M NaCl draw solution. RO: flat sheet, TFC, polyamide Flux: 4–8 L·m^−2^·h^−1^	Glucose: 100 mg·L^−1^ Peptone: 100 mg·L^−1^ KH_2_PO_4_: 17.5 mg·L^−1^ MgSO_4_: 17.5 mg·L^−1^ FeSO_4_: 10 mg·L^−1^ CH_3_COONa: 225 mg·L^−1^ Urea: 35 mg·L^−1^ 31 TrOCs: 5 μg·L^−1^ for each TrOC	The system achieved the effective removal of bulk organic matter, nutrients and almost complete removal of all 31 trace organic contaminants investigated	Not mentioned	Not mentioned	[190,207]
NF and/or RO (Semi-batch, 60 d)	Microfiltered, biologically treated sewage effluent	MF: 0.14 μm, flat sheet NF: 700 Da, flat sheet, 4 bar RO: 100 Da	pH: 6.8–7.6 EC: 520–1120 μS·cm^−1^ DOC: 3.6–7.7 mg·L^−1^ SAR: 39 F^−^: 0.7–1.1 mg·L^−1^ Cl^−^: 150–300 mg·L^−1^ NO_3_^−^: 1.0–1.3 mg·L^−1^ PO_4_^3–^: 0.74–0.99 mg·L^−1^ SO_4_^2−^: 49–51 mg·L^−1^ Na^+^: 81–120 mg·L^−1^ K^+^: 15–21 mg·L^−1^ Ca^2+^: 21–40 mg·L^−1^ Mg^2+^: 10–15 mg·L^−1^ BO_3_^3−^: 0.04–0.06 mg·L^−1^	Blending NF permeate and RO permeate at ratio of 50: 50. DOC: 95 ± 2% (NF), > 99% (RO) SAR: 8 Na^+^: 57 mg·L^−1^ Cl^−^: 109 mg·L^−1^ Ca^2+^: 7 mg·L^−1^ Mg^2+^: 5 mg·L^−1^ K^+^: 6 mg·L^−1^ S: 0.5 mg·L^−1^ NO_3_^−^: 7 mg·L^−1^ B: <0.1 mg·L^−1^ PPCPs (12 types): < 57 ng·L^−1^ per PPCP with NF; all < 5 ng·L^−1^ per PPCP with RO, except caffeine 39 ng·L^−1^	Irrigation	Australian and New Zealand Guidelines for Fresh and Marine Water Quality	[88]
RO	MBR effluent from domestic wastewater	TMP: 4–12 bars Initial Permeability: 3.6 L·m^−2^·d^−1^·bar^−1^	UV_254 nm_: 0.148–0.155 UV_210 nm:_ 1.579–3.207 TOC: 6.0–8.0 mg·L^−1^ COD: 12–13 mg·L^−1^ TSS: < 2 mg·L^−1^ Mg^2+^: 8.9–9.8 mg·L^−1^ Ca^2+^: 25.0–28.4 mg·L^−1^ EC: 631–894 μS·cm^−1^	UV_254 nm_ > 95% UV_210 nm:_ 90–97% TOC: 91–98% EC: 96–98%	Not mentioned	Not mentioned	[166]
MBR/MF + RO	Degritted sewage	Flow rate: MBR: 40 m^3^·d^−1^; MF: 0.4 μm RO: 19 m^3^·d^−1^	TSS: 201 mg·L^−1^ BOD_5_: 198 mg·L^−1^ COD: 391 mg·L^−1^ TKN: 43 mg·L^−1^ Nitrate N: 0.2 mgN·L^−1^ TDS: 337 mg·L^−1^ pH: 7.2 Color: 133 Hazen Alkalinity: 179 mg·L^−1^ Si: 12 mg·L^−1^ Turbidity: 59 NTU *E. coli*: 4.1 × 10^7^CFU·100 mL^−1^ Virus: 6.2x10^4^ PFU·100 mL^−1^ Total *Estrogens*: 182 μg·L^−1^	TSS: < 2 mg·L^−1^ BOD_5_: < 2 mg·L^−1^ COD: < 2 mg·L^−1^ TKN: 0.1 mg·L^−1^ Nitrate N: 0.9 mgN·L^−1^ TDS: 42 mg·L^−1^ pH: 5.4 Color: < 1 Hazen Alkalinity: 5.1 mg·L^−1^ Si: 0.5 mg·L^−1^ EC: 27 μS·cm^−1^ *E. coli*: nd Virus: nd Total *Estrogens*: 4.7 μg·L^−1^	Both for potable and non-potable reuse	USEPA and WHO guidelines	[118]
MF+RO	Secondary treated effluent from sewage	MF: 26 m^3^·d^−1^; 0.4 μm RO: 19 m^3^·d^−1^	TSS: 2 mg·L^−1^ BOD_5_: 3 mg·L^−1^ COD: 23 mg·L^−1^ TKN: 3.1 mg·L^−1^ Nitrate N: 4.7 mgN·L^−1^ TDS: 364 mg·L^−1^ pH: 7.2 Color: 44 Hazen Alkalinity: 71 mg·L^−1^ Si: 11.7 mg·L^−1^ Turbidity: 0.6 NTU *E. coli*: 2.8 × 10^5^ CFU·100 mL^−1^ Virus: 97 PFU·100 mL^−1^ Total *Estrogens*: 38 μg·L^−1^ Odor: 2	TSS: < 2 mg·L^−1^ BOD_5_: < 2 mg·L^−1^ COD: < 2 mg·L^−1^ TKN: 0.3–0.4 mg·L^−1^ Nitrate N: 0.71–1.43 mgN·L^−1^ TDS: 17–24 mg·L^−1^ pH: 5.3–5.5 Color: < 2.5 Hazen Alkalinity: 2.7–3.3 mg·L^−1^ Si: 0.3–0.7 mg·L^−1^ EC: 24–33 μS·cm^−1^ *E. coli*: nd Virus: nd Total *Estrogens*: < 4.4 μg·L^−1^ Odor: 1	Both for potable and non-potable reuse	EPA and WHO guidelines	[118]
MBR+RO (pilot scale, 112 days)	Primary municipal wastewater	UF: 0.04 μm, hollow fiber.TMP: 0.42 bar (UF), 15.2 bar (RO)	TSS: 100–1930 mg·L^−1^ Turbidity: 7–308 NTU COD: 122–2205 mg·L^−1^ DOC: 2.12–10.21 mg·L^−1^ (after MBR) UV_254_: 0.30–4.00 TN: 12.6–205 mg·L^−1^ Pb: 1–16 μg·L^−1^ Ni: 1–33.7 μg·L^−1^ Cu: 1–1345 μg·L^−1^ Cr: 1–746 μg·L^−1^	TSS: <1 mg·L^−1^ Turbidity: 0.01–0.13 NTU COD: < 32 mg·L^−1^ DOC: 1.04–4.1 mg·L^−1^ UV_254_: 0.001–0.01 TN: 17–21 mg·L^−1^ Pb: < 1 μg·L^−1^ Ni: < 1 μg·L^−1^ Cu: < 1 μg·L^−1^ Cr: < 1 μg·L^−1^	Not mentioned	Not mentioned	[208]
AnMBR+RO (lab scale)	Synthetic municipal wastewater	Flux: 20 L·m^−2^·h^−1^	COD: 400 mg·L^−1^ NH_4_^+^-N: 45 mg·L^−1^ PO_4_^3−^-P: 5 mg·L^−1^ NaHCO_3_: 500 mg·L^−1^ CaCl_2_·2H_2_O: 45 mg·L^−1^ MgSO_4_·7H_2_O: 20 mg·L^−1^ FeSO_4_·7H_2_O: 20 mg·L^−1^ FeCl_3_·7H_2_O: 1.5 mg·L^−1^	Ammonium-N: 2.1 mg·L^−1^ Phosphate–P: 0.03 mg·L^−1^ TOC: 0.13 mg·L^−1^ Sodium: 3.2 mg·L^−1^ Potassium: 0.084 mg·L^−1^ Calcium: 0.05 mg·L^−1^ Iron: < 0.005 mg·L^−1^ Chloride: 4.7 mg·L^−1^ Sulfate: 0.5 mg·L^−1^ Conductivity: 47 μS·cm^−1^	Discharge to reservoirs for indirectly potable reuse	Guidelines for NEWater in Singapore	[185]
AnMBR + RO (lab scale)	Simulation of municipal wastewater	UF: 0.02 μm	Sucrose: 210 mg·L^−1^ Meat extract: 41.7 mg·L^−1^ Peptone: 60 mg·L^−1^ NH_4_Cl: 95.5 mg·L^−1^ KH_2_PO_4_: 22 mg·L^−1^ CaCl_2_·2H_2_O: 10 mg·L^−1^ FeSO_4_·7H_2_O: 10 mg·L^−1^ MgSO_4_·7H_2_O: 10 mg·L^−1^ NaHCO_3_: 400 mg·L^−1^	TOC: 0.17 ± 0.02 mg·L^−1^ NH_4_–N: 0.81 ± 0.09 mg·L^−1^ Phosphate–P: < 0.01 mg·L^−1^ Na^+^: 2.30 ± 0.12 mg·L^−1^ K^+^: 0.16 ± 0.01 mg·L^−1^ Ca^2+^: 0.04 ± 0.01 mg·L^−1^ Mg^2+^: < 0.01 mg·L^−1^ Iron:< 0.01 mg·L^−1^ Aluminum:< 0.01 mg·L^−1^ Chloride: 2.35 ± 0.50 mg·L^−1^ Sulfate: 0.06 ± 0.01 mg·L^−1^ EC: 24.19 ± 1.21 μS·cm^−1^	NEWater product	Guidelines for NEWater in Singapore	[145]
NF–MBR+RO	Municipal wastewater	NF: 200–300 Da Flux: 10 L·m^−2^·h^−1^ MLSS: 513 ± 96 mg·L^−1^	COD: 389.8 ± 169.9 mg·L^−1^ DOC: 48.3 ± 13.9 mg·L^−1^ Ca^2+^: 28.9 ± 3.5 Mg^2+^: 7.9 ± 1.2 mg·L^−1^ Na^+^: 105.2 ± 6.9 mg·L^−1^ TN: 40.7 ± 6.1 NH_4_–N: 40.9 ± 4.5 mg·L^−1^ NH_3_–N: nd PO_4_^3−^: 23.6 ± 3.2 mg·L^−1^ EC: 859.5 ± 18.9 mS·cm^−1^	NF permeate: COD: 99.6 ± 0.8% DOC: 0.5–2.5 mg·L^−1^, 97.5% ± 1.8% Biopolymer: nd Humic substances: 0.1 mg·L^−1^	Not mentioned	Not mentioned	[184]

SAR: sodium adsorption ratio; PPCPs: pharmaceuticals and personal care products; TKN: total Kjeldahl nitrogen; FAO: Food and Agriculture Organization; TrOCs: trace organic contaminants.

**Table 10:** General effectiveness of commonly used disinfection processes [212,239].

Disinfection Method			Effectiveness of Inactivation			By Products	Secondary Disinfection *	Stability
			Bacteria	Viruses	Protozoa			
Chemical	Chlorination	**Chlorine Cl_2_**	++++	++++	+	Trihalomethanes, Haloacetic acids, Haloacetonitriles, Haloketones, Chloral hydrate, Chloropicrin, Cyanogen chloride, Chlorate, Chloramines	++++	stable
		Sodium hypochlorite (NaClO)						
		Chloramines	+++	++	+	Dichloramines, Trichloramines, Cyanogen chloride, Chloral hydrate	++++	stable
		Chlorine dioxide (ClO_2_)	++++	++++	+++	Chlorites, Chlorates, Chlorides	+++	unstable
	Ozonation		++++	++++	+++	Bromate Aldehydes Ketones Ketoacids Carboxylic acids Bromoform Brominated acetic acids	–	unstable
Physical	UV		+++	++	++++	–	–	unstable

* Secondary disinfection: keep disinfection status in the water distribution system to prevent regrowth of pathogens. ++++: excellent; +++: good, ++: moderate; +: poor; -: no or non-function

**Table 11:** Combination of membrane processes with disinfection processes for wastewater reuse applications.

Process	Feed for Disinfection	Performance of Disinfection	Applications	References
MBR + Chlorination	Secondary effluent of municipal WWTP	Chlorination helps to inactive bacteria and residual viruses from MBR. Compared to the MBR permeate, effluent after chlorination stands out at: complete removal on thermo-tolerant coliforms, *E. coli, Enterococci*, F-RNA specific bacteriophages and bacteriophages infecting Bacteroides fragilis; Increasing removal efficiency on total coliforms, Fecal coliforms and Somatic coliphages for which LRV are 1, 0.6 and 1.5, respectively, compared to MBR permeate.	urban (e.g., street cleaning, vehicle washing) and agricultural reuse.	[125]
MBR + UV	Raw Sewage	UV disinfection is proposed to provide an extra barrier for removal of pathogens, ensuring high-quality effluent standards. The hybrid process showed a high removal efficiency (90%) on most trace organic chemical contaminants.	Agriculture reuse	[240]
Membrane filtered process + sequential chlorination	Tertiary effluent of municipal WWTP	Sequential chlorination is beneficial to optimize free chlorine (virus and N-nitrosodimethylamine control) and chloramine disinfection (trihalomethane, haloacetic acid and coliform control). The increase of chlorine residual and the contact time also increased the formation of unregulated halogenated DBP classes.	Direct potable reuse	[215]
MBR + sequential UV/chlorine	Tertiary effluent of municipal WWTP	Sequential UV/chlorine processes with a suitable dose of disinfectants decreased microorganism concentrations below detection limits, including heterotrophic plate count, total bacteria count and total coliforms. The disadvantage is the byproducts produced by disinfection (60.2 ug·L^−1^ Trihalomethanes).	Reclaimed water	[241]
Ozonation + MF	Secondary effluent from municipal WWTP	Ozone helps to improve removal efficiency on color, COD, TN and turbidity in wastewater and lower fouling potential on MF, but with less impact on TP removal. Feed water quality:pH: 7.2 ± 0.61; COD: 35.0 ± 8.15 mg·L^−1^ Turbidity: 1.53 ± 1.82 NTU; SS: 7.1 ± 5.9 mg·L^−1^; UV_254_: 0.095 ± 0.021 cm^−1^; DOC: 6.29 ± 1.53 mg·L^−1^; Color: 30 ± 4 CU; T–P: 2.98 ± 1.68 mg·L^−1^; T–N: 11.1 ± 3.4 mg·L^−1^ Permeate: pH: 7.6; COD: 14–25 mg·L^−1^; Turbidity: 0.61–0.87 NTU; Color: 2–3 CU; TP: 2.67–2.84 mg·L^−1^; TN: 6.2–7.9 mg·L^−1^	Wastewater reuse proposed by South Korean Ministry of Environment	[242]
Ozone + biologic activated carbon (BAC) + MF +UF	Secondary effluent from municipal WWTP	The combined O_3_/BAC/MF/RO train was effective for eliminating N-nitrosamines and the total toxicity-weighted byproduct precursor concentrations	Potable reuse	[243]
Sedimentation + UV/peracetic acid (PAA) /ozone (O_3_)	Secondary effluent from municipal WWTP in Italy	All three disinfectants can provide qualified effluent for irrigation, according to WHO, with enough contact time and quantity. Through comparison, UV physical disinfection showed extremely fastest kinetics with contact time <20 sec.	Irrigation proposed by WHO guidelines	[220]

**Table 12:** Combination of membrane processes with advanced oxidation processes (AOPs) for wastewater reuse applications.

Process	Feed for Disinfection	Performance of AOPs	Applications	References
UV/S_2_O_8_^2−^ or UV/H_2_O_2_	RO concentrate in municipal wastewater recycling facilities in California	◇ Both AOPs were effective in significantly removing four pharmaceuticals with 250 mJ·cm^−2^ average fluence requirement. Specially, UV/S_2_O_8_^2−^ provides significantly better performance treatment for some constituents. ◇ In the UV/H2O2 process, effluent organic matter could decrease ~75% of the OH•, reducing ~75% degradation efficiency on the target contaminants. ◇ In UV/S_2_O_8_^2−^ process, although effluent organic matter decreased ~93% of the SO_4_^2−^, because daughter radicals Cl^−^ contributed to contaminant degradation, the reduction in contaminant degradation efficiency was only ~75–80%.	Discharge	[244]
Biologic media filtration + O_3_ + UV + Chlorination	Secondary treated wastewater	◇ The whole process showed wide and high removal efficiency on various compounds (Nitrosamines, Endocrine-disrupting chemicals, pharmaceuticals, herbicides, perfluorinated compounds, etc.), which achieved more than 69% of total removal of most compounds (387 specific analytes). ◇ Ozone significantly increased the degradation rate on most contaminants. ◇ However, ozonation caused some contaminant increases (N-nitrosodimethylamine, bisphenol, desisopropyl atrazine) in the effluent and chlorination produced related byproducts (Trihalomethanes, desmethyl atrazine, chloral hydrate).	Reuse for irrigation, dual recirculation and fire fighting	[245]
MF + RO + UV/H_2_O_2_	Secondary effluent from conventional wastewater treatment facility	◇ UV/H_2_O_2_ process degraded the remaining low-molecular-weight organic compounds and micropollutants. ◇ UV-AOP increased the 6-log of virus removal credits after RO treatment. ◇ The operation MF+RO+UV/H_2_O_2_ process accounted for about 70–75% energy demand and 75–80% CO_2_ emission compared to a UF osmosis MBR system, respectively, with the positive relationship with disposal pressure.	Potable reuse, Recharge to groundwater	[232]
MF + RO + UV/H_2_O_2_	secondary municipal wastewater	◇ UV/H_2_O_2_ provides good water quality for reuse, which significantly removed over 98% of each tested micropollutant, including N-nitrosodimethylamine, endocrine disrupting compounds, herbicides, pesticide (Metaldehyde)… ◇ MF/RO+ UV/H_2_O_2_ was shown to be the most cost-effective process, with around 20% lower operational expenditures than MF- UV/H_2_O_2_ without requirement in RO concentrate disposal.	Potable reuse	[233]
MF + Ozonation	Treated greywater	◇ The oxidation process improved the removal efficiency of *E. coli,* total coliform, *Salmonella and Staphylococcus*, from 30% after MF, to 100% after ozonation. ◇ All pathogenic microorganisms were removed at the contact time range of 0–15 min with O_3_	Firefighting, plants irrigation, toilet flushing and car washing	[246]

**Table 13:** Wastewater recovery rate and energy consumption of membrane-based technologies.

Process	Productivity	Membrane Flux	Recovery Rate	Energy Consumption	References
GAC + MF		30 L·m^−2^·h^−1^	98%		[100]
UF (pilot plant, 2 years)	< 0.7 m^3^·h^−1^	17–22 L·m^−2^·h^−1^			[81]
UF (pilot plant, two months)	7.7–9.0 m^3^·h^−1^	330–380 L·m^−2^·h^−1^	82.3–96.5% (without chemical cleaning)		[77]
UF	1.0 m^3^·h^−1^	28.5 L·m^−2^·h^−1^	About 85% (without chemical cleaning)		[109]
MBR + disinfection		21.5 L·m^−2^·h^−1^		<0.5 kWh ·m^−3^ (with productivity > 15 m^3^·d^−1^)	[149]
FO + NF	0.16 (FO) and 0.187–0.35 (NF) m^3^·h^−1^	FO: 2.4 L·m^−2^·h^−1^; NF: 3.3 or 6.6 L·m^−2^·h^−1^		3.44–4.57 kWh ·m^−3^	[197]
RO	0.79 m^3^·h^−1^		65–75% (RO itself)		[118]
RO	0.04 m^3^·h^−1^		50% (RO itself)		[208]
NF–MBR + RO		MBR permeate flux: 10 L·m^−2^·h^−1^; RO permeate flux: 20 L·m^−2^·h^−1^	90%	0.739 kWh·m^−3^	[184]
UF–MBR + RO		MBR permeate flux: 10 L·m^−2^·h^−1^; RO permeate flux: 20 L·m^−2^·h^−1^	75%	0.732 kWh·m^−3^	[184]
MBR + RO				0.518 kWh ·m^−3^	[145]
AnMBR + RO		AnMBR permeate flux: 10–20 L·m^−2^·h^−1^ RO permeate flux: 20 L·m^−2^·h^−1^	RO: 75% (RO itself)	0.333 kWh ·m^−3^ (consider energy recovery with methane)	[145]
AnMBR + RO + IE		MF permeate flux: 10 L·m^−2^·h^−1^ RO: 20 L·m^−2^·h^−1^	RO: 75% (RO itself)	0.915 kWh·m^−3^ (total consumption); 0.368 kWh·m^−3^ (consider energy recovery with methane)	[185]

**Table 14:** Advantages and disadvantages of various membrane-based treatment processes.

Treatment Processes	Reuse Level	Advantages	Disadvantages
MF/UF-based treatment	Non-potable reuse: toilet flushing, urban uses, irrigation, etc.	• Low-pressure driven process, high flux and high permeability. • Low energy cost • Effective removal of high molecular weight matters, bacteria and viruses. • UF can almost eliminate all the bacteria, protozoa and viruses, compared to MF. • MF/UF membranes pretreated with physical/chemical process results in low fouling potential, long term operation and higher load rate.	• Health risks potential for humans. • Incomplete removal of low molecular weight matters, dissolved organics, salinity and micropollutants, etc. • Fouling potential
MBRs	Non-potable reuse: Toilet flushing, cleaning, process water, urban uses, irrigation, etc.	• Low-pressure driven process, high flux and high permeability. • Low energy cost • Effective removal of organics, TSS, nutrients like N, P, S in various forms, surfactants and micropollutants from various wastewater with biologic process. • Less organic foulants on the membrane, smaller footprint, faster plant activation, no biologic sedimentation units and less sludge production compared to CAS process. • Particularly, AnMBRs hold significant potential to reduce the overall energy demand, together with resource recovery	• Health risks potential for humans. • Incomplete removal of low molecular weight matters, dissolved organics, salinity and micropollutants, etc. • Fouling potential with membrane pore-clogging and sludge cake deposition
NF/RO-based treatment	Non-potable and potable reuse: agricultural irrigation, groundwater recharge, indirect potable water, etc.	• High removal efficiency of micropollutants, microorganisms and salinity, EC, other dissolved organic and inorganic matters. • Reduce human health concerns • High level of reuse applications	• High-pressure driven process, with low flux and low permeability. • High energy cost. • Pretreatment demand • Biofouling caused by microorganisms.
FO-based treatment	Non-potable and potable reuse: agricultural irrigation, groundwater recharge, indirect potable water, etc.	• FO: non external pressure driven process • Lower membrane fouling potential than RO due to less formation and compaction of cake layers on FO membranes in the absence of hydraulic pressure • High flux recovery after cleaning and high water recovery using low-grade energy resources • Combination of municipal wastewater treatment with seawater desalination	• FO is a process of dilution, which needs further separation treatment. • High requirements for the selection of draw solution and membrane material
Disinfection/AOPs combined with membrane process	Non-potable or potable reuse based on the membrane types	• Disinfection can always act as the last step to ensure water quality and disinfection for human health. • Disinfection absolutely eliminates microorganisms. • Long-term effects of disinfectants, such as chlorine. • AOPs absolutely eliminates organic pollutions, micropollutants and microorganisms. • Improvement of water reuse levels when combined with MF/UF/MBRs.	• Formation of harmful byproducts both from disinfection and AOPs. • High chemical cost and unstable storage of chemicals in AOPs
